# The WUSCHEL-related homeobox transcription factor *CsWOX3* negatively regulates fruit spine morphogenesis in cucumber (*Cucumis sativus* L.)

**DOI:** 10.1093/hr/uhae163

**Published:** 2024-06-14

**Authors:** Shuo Xu, Yaru Wang, Songlin Yang, Shanshan Fan, Kexin Shi, Fang Wang, Menghang An, Yu Qi, Mingqi Wang, Min Feng, Zhifang Li, Xingwang Liu, Huazhong Ren

**Affiliations:** Engineering Research Center of Breeding and Propagation of Horticultural Crops, Ministry on Education, College of Horticulture, China Agricultural University, Beijing 100193, China; Engineering Research Center of Breeding and Propagation of Horticultural Crops, Ministry on Education, College of Horticulture, China Agricultural University, Beijing 100193, China; Sanya Institute of China Agricultural University, Sanya Hainan 572000, China; Engineering Research Center of Breeding and Propagation of Horticultural Crops, Ministry on Education, College of Horticulture, China Agricultural University, Beijing 100193, China; Engineering Research Center of Breeding and Propagation of Horticultural Crops, Ministry on Education, College of Horticulture, China Agricultural University, Beijing 100193, China; Engineering Research Center of Breeding and Propagation of Horticultural Crops, Ministry on Education, College of Horticulture, China Agricultural University, Beijing 100193, China; Engineering Research Center of Breeding and Propagation of Horticultural Crops, Ministry on Education, College of Horticulture, China Agricultural University, Beijing 100193, China; Engineering Research Center of Breeding and Propagation of Horticultural Crops, Ministry on Education, College of Horticulture, China Agricultural University, Beijing 100193, China; Engineering Research Center of Breeding and Propagation of Horticultural Crops, Ministry on Education, College of Horticulture, China Agricultural University, Beijing 100193, China; Engineering Research Center of Breeding and Propagation of Horticultural Crops, Ministry on Education, College of Horticulture, China Agricultural University, Beijing 100193, China; Engineering Research Center of Breeding and Propagation of Horticultural Crops, Ministry on Education, College of Horticulture, China Agricultural University, Beijing 100193, China; Sanya Institute of China Agricultural University, Sanya Hainan 572000, China; Engineering Research Center of Breeding and Propagation of Horticultural Crops, Ministry on Education, College of Horticulture, China Agricultural University, Beijing 100193, China; Engineering Research Center of Breeding and Propagation of Horticultural Crops, Ministry on Education, College of Horticulture, China Agricultural University, Beijing 100193, China; Sanya Institute of China Agricultural University, Sanya Hainan 572000, China; Engineering Research Center of Breeding and Propagation of Horticultural Crops, Ministry on Education, College of Horticulture, China Agricultural University, Beijing 100193, China; Sanya Institute of China Agricultural University, Sanya Hainan 572000, China

## Abstract

Cucumber (*Cucumis sativus* L.) is a widely cultivated crop with rich germplasm resources, holding significant nutritional value. It also serves as an important model for studying epidermal cell fate and sex determination. Cucumbers are covered with multicellular and unbranched trichomes, including a specific type called spines found on the surface of the fruit. The presence and density of these fruit spines determine the visual quality of cucumber fruits. However, the key regulatory genes and mechanisms underlying cucumber fruit spine development remain poorly understood. In this study, we identified a WUSCHEL-related homeobox (WOX) family gene *CsWOX3*, which functioned as a typical transcriptional repressor and played a negative role in fruit spine development. Spatial–temporal expression analysis revealed that *CsWOX3* exhibited a relatively high expression level in the cucumber female floral organs, particularly in the fruit exocarp. Knockout of *CsWOX3* using CRISPR/Cas9 resulted in a significant 2-to-3-fold increase in the diameter of fruit spines base, while overexpression led to a 17% decrease in the diameter compared to the wild-type. A SQUAMOSA PROMOTER BINDING PROTEIN-LIKE transcription factor CsSPL15 could directly bind and activate the expression of *CsWOX3*, thereby suppressing the expression of downstream auxin-related genes, such as *CsARF18*. Additionally, the RING-finger type E3 ubiquitin ligase CsMIEL1-like interacted with the HD domain of CsWOX3, which might result in the ubiquitination and subsequent alteration in protein stability of CsWOX3. Collectively, our study uncovered a WOX transcription factor *CsWOX3* and elucidated its expression pattern and biological function. This discovery enhances our comprehension of the molecular mechanism governing cucumber fruit spine morphogenesis.

## Introduction

Plant trichomes are hair-like epidermal structures that widely cover the surface of most vascular plants and are formed exceptionally through the polar growth of plant epidermal cells [1–5]. Trichomes come in diverse morphologies and structures, categorized as either unicellular and multicellular, or non-glandular and glandular based on their secretory capacity [5–7]. Functionally, plant trichomes act as a natural mechanical barrier, protecting plants from external damage [5, 8]. In addition to this primary function, they also play various other important roles. For example, the cotton fibers’ trichomes found on the seed epidermis hold significant economic value. Glandular trichomes in tomatoes can produce terpene secretory substances used in the production of insecticides. Another noteworthy example is Artemisinin, a terpenoid ester secreted by *Artemisia annua* glandular trichomes, renowned for its medicinal properties [7, 9–13].

Cucumber (*Cucumis sativus* L.), a vegetable crop belonging to the Cucurbitaceae family, is extensively cultivated worldwide [2, 4, 14, 15]. Unlike the branched unicellular trichomes in the model plant *Arabidopsis thaliana*, cucumber trichomes are non-branched and multicellular, and widely found in different parts of the cucumber plant, including leaves, stems, flowers, tendrils, fruits, *etc* [5, 16–19]. Among them, the trichomes distributed on the surface of cucumber fruits are also known as fruit spines, and which presence and density are significant factors in determining the visual quality of the fruits, making it an important characteristic for commercial purposes [2, 5, 18–21]. The common fruit spines, consisting of a spherical or conical base and a sharp stalk, sometimes develop on the tubercules, which are known as the fruit warty trait and hold great commercial value for cucumber fruits [18, 19, 21].

Several regulatory genes and endogenous hormone signals have been identified to play pivotal roles in the development of cucumber fruit spines. Dong *et al.* [5] observed that the trichomes on the surface of cucumber cotyledons seemed to share a time-course developmental process with fruit spines, and their process unfolded into five stages: initiation (I), first division (II), glandular trichomes head transition/non-glandular trichomes tip head formation (III), glandular head formation/non-glandular trichomes elongation (IV), and glandular trichomes active metabolism/formation of non-glandular trichomes multicellular base (V). Notably, A HD-ZIP IV transcription factor *CsTril*/*CsGL3* plays a critical role in the fate determination and initial development of cucumber trichomes (stage I), and their mutants exhibit a complete absence of trichome on various parts of the plant, including stems, leaves, tendrils, fruits, *etc* [5, 17, 20, 22]. Another critical transcription factor, *CsGL1*/*CsMict*/*CsTBH*, is a member of the HD-Zip I family and plays a role in trichome development at stage III [2, 17, 20, 23–26]. It functions as a downstream factor of *CsTril*/*CsGL3*, and the loss of its bio-function results in the formation of stunted trichomes. Recent studies have brought to light several novel regulatory factors involved in the development of cucumber fruit spines. Among them, the cucumber hard spines gene CsTs, a C-type lectin receptor-like tyrosine protein kinase, plays a crucial role in tender fruit spine formation and interacts with the auxin signaling factor CsVTI11 to influence cell polarity and spine development [27, 28]. *CsNS*, a member of the AUX1/LAX family, is specifically expressed in the fruit peel and spines and regulates the density of fruit spines through the auxin responsive Aux/IAA family genes independently of the *CsTu* [29, 30]. Furthermore, Guo *et al.* [31] have demonstrated that the cucumber APETALA2/ETHYLENE RESPONSE FACTOR (AP2/ERF) CsTOE3 directly interacts with CsGL1 and CsTTG1 to control the development of type II non-glandular fruit trichomes partly through a specific ethylene-related pathway. CsSBS1, a C2H2 zinc finger transcription factor, positively regulated the development of cucumber fruit spines base, and could also form a trimer complex with CsTTG1 and CsGL1 to regulate the size of cucumber fruit spines base through the ethylene signal pathway [26]. Although several regulatory genes have been reported in various development stages, the molecular mechanisms underlying cucumber fruit spine development remain incompletely understood. Therefore, it is essential to explore more novel regulatory factors to enhance our comprehension of the intricate molecular network in the future.

To further investigate key genes in fruit spine development, we identified *CsWOX3*, a member of the WUSCHEL-related homeobox (WOX) transcription factor in cucumber. *CsWOX3* is a homolog of *OsWOX3B,* known for its involvement in governing the initiation and elongation of rice trichomes [32–36]. *CsWOX3* acted as a transcriptional repressor and negatively regulated the morphogenesis of cucumber fruit spines. Additionally, we identified the *CsSPL15*-*CsWOX3*-IAA/AUX transcriptional regulatory module and confirmed the physical interaction between CsWOX3 and the RING-finger type E3 ubiquitin ligase CsMIEL1-like during cucumber spine development. In conclusion, our study uncovers CsWOX3 as a novel transcriptional regulator that negatively impacts the morphogenesis of fruit spines in cucumber, which occurs through the auxin- and ubiquitin-mediated pathway. This study contributes to enriching the molecular mechanism of cucumber trichome development.

## Results

### CsWOX3 functions as a typical WOX family transcriptional repressor

**Figure 1:**
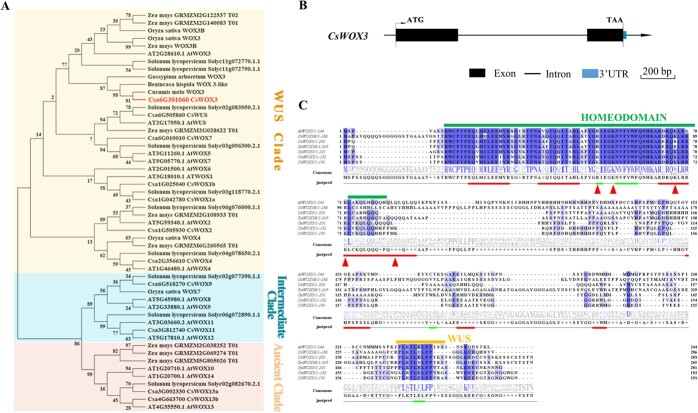
Phylogenetic relationship, gene structure, and protein domains of CsWOX3. **A** Phylogenetic analysis using maximum likelihood between all cucumber WOX proteins and selected WOX proteins from other species. CsWOX3 (marked in red) exhibits a homologous relationship with OsWOX3B. They also belong to the WUS subclass of the WOX family together with AtWOX3 in *Arabidopsis*, OsWOX3 in rice, ZmWOX3A/B in maize, and SlWOX3A/B in tomato, *etc*. **B** Gene structure diagram of *CsWOX3*. *CsWOX3* is located on Chromosome 6, with a gene length of 1366 bp and a CDS length of 582 bp, including two exons and one intron. **C** Protein sequence and domain analysis of CsWOX3. The N-terminus of CsWOX3 features a conserved HOMEODOMAIN (HD) domain (marked in green), while the C-terminus region includes a WUS-box protein motif with the sequence ‘TLRTLELFPV’ (marked in yellow). Notably, there is an absence of a typical acidic region or EAR motif. Furthermore, the red triangles highlight the predicted ubiquitination modification sites.

**Figure 2:**
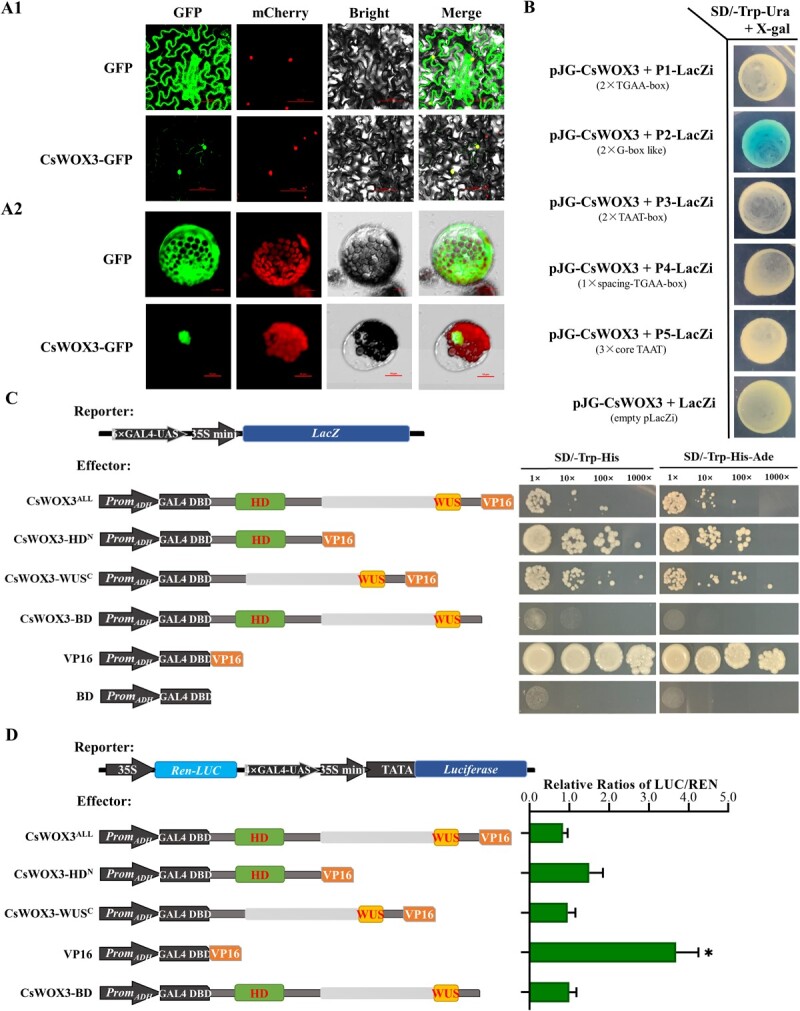
CsWOX3 is a typical WOX transcriptional repressor. **A** Subcellular localization of CsWOX3-GFP in *Nicotiana benthamiana* leaf cells 60 h post inoculation (A1, bar = 100 μm) and cucumber protoplasts 20 h post transfection (A2, bar = 10 μm). **B** Verification of downstream DNA *cis-*elements bound by CsWOX3 using yeast one-hybrid LacZi reporter system. **C** Validation of transcriptional activation activity of different CsWOX3 domain truncations fused with VP16 protein in yeast. Left: Schematic diagram of vectors with different protein domain truncations. Right: The result of transcriptional activity of different CsWOX3 domain truncations in yeast. **D** Transient infection of *Nicotiana benthamiana* to examine the transcriptional inhibition efficiency of different CsWOX3 domain truncations. Left: Schematic diagram of different vectors. Right: The relative ratio of firefly luciferase (LUC) to renilla luciferase (REN). Error bars represent *SD* from three biological replicates; ^*^P < 0.05.

*OsWOX3B*, a WUSCHEL-related homeobox (WOX) transcription factor, is crucial for controlling rice development, and its mutation results in reduced or complete absence of trichomes on leaves and seed glumes [32–36]. Through phylogenetic analysis in cucumber, we identified CsWOX3 (Csa6G301060) as the homolog of OsWOX3B (Fig. 1A). CsWOX3 belonged to the WUS sub-family, as evidenced by the conserved HOMEODOMAIN (HD) domain and WUS domain (Fig. 1B–C). Then we further characterized CsWOX3 as a member of the WOX family transcription factors by analysing its subcellular localization, transcriptional activation activity and interaction with downstream DNA-binding *cis*-elements (Fig. 2). We observed that CsWOX3 localized to the nucleus after fusion with GFP protein and specifically bound to the G-box like DNA *cis*-element, which contained the conserved core sequence TCACGTGA (Fig. 2A–B). Additionally, when CsWOX3 was fused with VP16 protein, it significantly inhibited the transcriptional activation activity of VP16, particularly through its C-terminal WUS domain (Fig. 2C–D). Collectively, these findings indicate that CsWOX3, the homolog of OsWOX3B, functions as a typical WOX family transcriptional repressor.

### Relatively high expression of *CsWOX3* within female floral ovary epidermis

**Figure 3:**
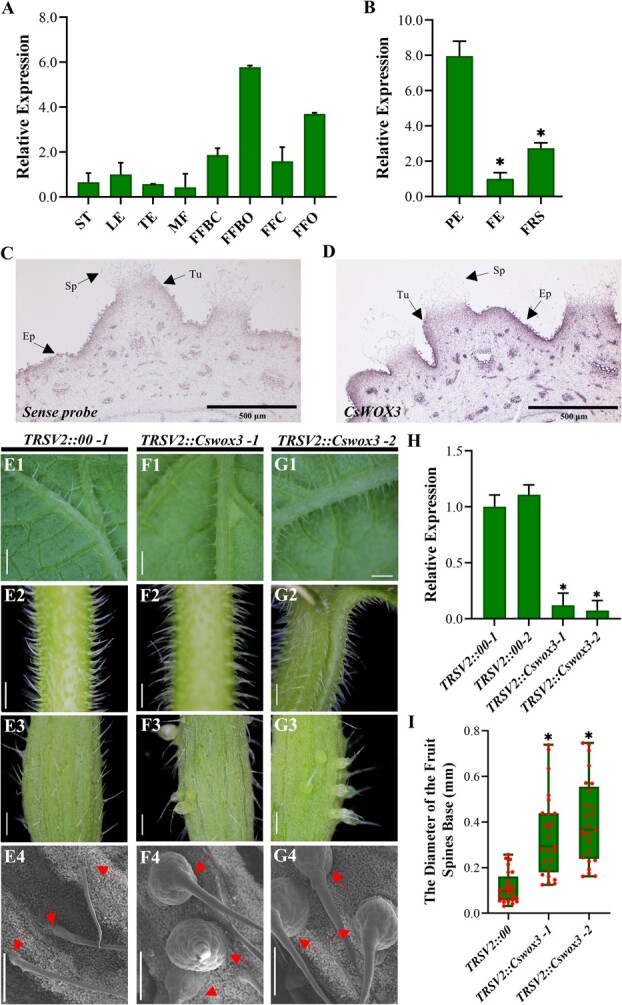
Expression pattern and preliminary study on the biological functions of *CsWOX3*. **A** The relative transcript level of *CsWOX3* in various cucumber tissues. FFBC, female flower bud corolla; FFBO, female flower bud ovary; FFC, female floral corolla; FFO, female floral ovary; LE, leaf; MF, male flower; ST, stem; TE, tendril. Error bars represent *SD* from 3 biological replicates. **B** The relative transcript level of *CsWOX3* within female floral ovary. FE, fruit flesh of the female floral ovary only; FRS, fruit spines upon the female floral ovary excluding the exocarp; PE, the exocarp of female floral ovary removing fruit spines while keeping glandular fruit trichomes. Error bars represent *SD* from three biological replicates; ^*^P < 0.05. **C** to **D** mRNA *in situ* hybridization of *CsWOX3* within female floral ovary in cucumber. Ep, epidermis; Sp, spines; Tu, tubercule; bar = 500 μm. The phenotype of trichomes attached to the surface of abaxial leaf (**E1**, **F1**, and **G1**), petiole (**E2**, **F2**, and **G2**) and ovary (**E3**, **F3**, and **G3**) within two independent *CsWOX3* TRSV-VIGS lines (*TRSV2::Cswox3–1* and *− 2*; bar = 1 mm). **E4**, **F4**, and **G4** present the SEM observation of cucumber fruit spines within *CsWOX3* TRSV-VIGS lines (Bar = 500 μm). Within 6101–4 wild-type plants, TRSV2 empty vector was used as a negative control (*TRSV2::00*), *TRSV2::Cspds* was used as a positive control. **H** The mRNA transcriptional level of *CsWOX3* within TRSV-VIGS lines. Significance compared to *TRSV2::00* was determined by Student’s *t* test; ^*^P < 0.05; N = 2, *n* = 3. **I** Average diameter of cucumber fruit spines base within TRSV-VIGS lines. Significance compared to *TRSV2::00* was determined by Student’s *t* test; ^*^P < 0.05; N = 2, *n* = 25.

Next, we investigated the spatial–temporal expression pattern of *CsWOX3*. Wild-type cucumbers 3661 were grown in pots until flowering and fruiting. We collected samples from various aboveground tissues and analysed the relative transcript level of *CsWOX3*. Our analysis revealed that *CsWOX3* was expressed in multiple cucumber tissues, including the stem, leaf, and tendrils. It is worth noting that its expression was relatively high in the female floral organs, with higher levels in the ovary but the corolla (Fig. 3A). Interestingly, the expression level was more pronounced in young female floral organs compared to those in later flowering stages. Further validation experiments confirmed that *CsWOX3* exhibited high expression in the exocarp without fruit spines (PE), while its expression in the fruit spines (FRS) and flesh (FE) was relatively low (Fig. 3B). Furthermore, mRNA *in situ* hybridization assay showed strong expression signals in the epidermis of the cucumber female floral ovary, whereas the expression signals in fruit spines and flesh were comparatively weak (Fig. 3C–D).

### *CsWOX3* negatively regulates the morphogenesis of cucumber fruit spines

**Figure 4:**
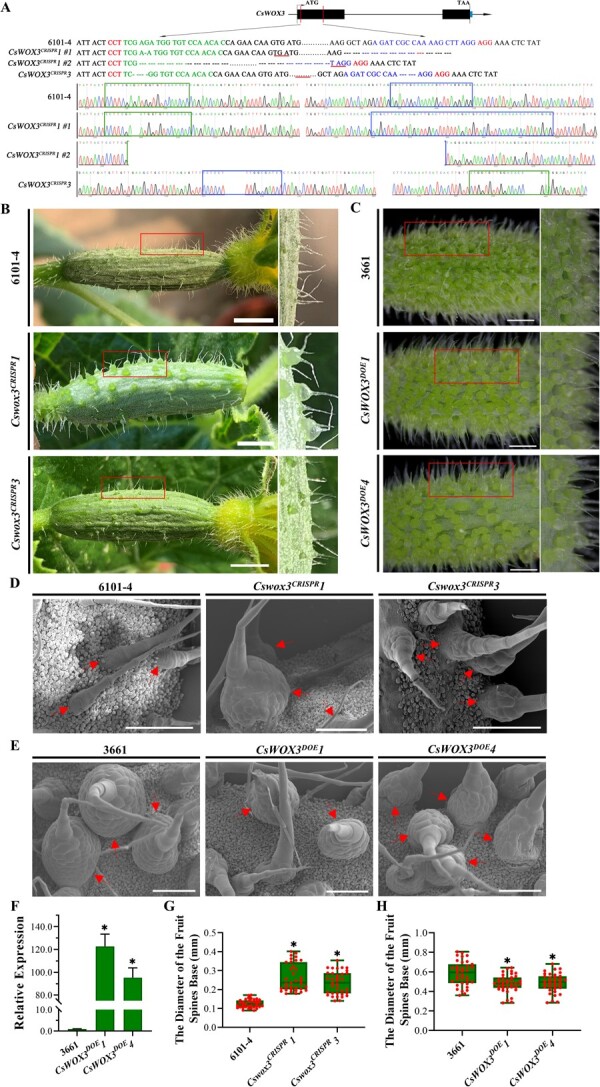
Critical role of *CsWOX3* in the morphogenesis of cucumber fruit spines. **A** Genetic modifications within the *CsWOX3* knockout (*Cswox3^CRISPR^*) lines. There are two types of genetic modifications in the *Cswox3^CRISPR^1* line: one involves 1 bp deletion from target 1 (highlighted in green) and a small fragment deletion from target 2 (marked in blue), while another type is the large fragment deletion between target 1 and target 2. In the *Cswox3^CRISPR^3* line, both target 1 and target 2 were deleted from the baseline. In addition, the base sequence marked in red is the PAM region. **B** to **C** Phenotypes of cucumber fruit spines within *CsWOX3* knockout (*Cswox3^CRISPR^*) (**B**, bar = 1 cm) and overexpression (*CsWOX3^DOE^*) (**C**, bar = 5 mm) lines. **D** to **E** Scanning electron microscope observation of spines attached on the female floral ovary within *CsWOX3* knockout (*Cswox3^CRISPR^*) (**D**) and overexpression (*CsWOX3^DOE^*) (**E**) lines, in comparison to their respective wild-type lines 6101–4 and 3661. Bar = 500 μm, and red arrows indicate these differences. **F** The relative transcript level of *CsWOX3* within overexpression (*CsWOX3^DOE^*) lines. Significance compared to WT was determined by Student’s *t* test; ^*^P < 0.05; *N* = 3. **G** to **H** Average diameter of cucumber fruit spines base was determined within CRISPR lines (G) and overexpression lines (H). Significance compared to WT was determined by Student’s *t* test; ^*^P < 0.05; *N* = 3.

To determine the biological function of *CsWOX3*, we initially silenced its transcription using the TRSV-VIGS system (Fig. 3E–I; Fig. S1A, see online supplementary material). A significant increase of over three times was observed in fruit spines base within *TRSV::Cswox3* silenced lines, while no significant differences were noted on the surface of leaves and petioles (or stems). This suggests that *CsWOX3* plays a central role in the development of cucumber fruit spines. Furthermore, we generated two *CsWOX3* knockout lines, *Cswox3^CRISPR^1* and *Cswox3^CRISPR^3*, using CRISPR/Cas9 gene editing technology (Fig. S1B–C, see online supplementary material). In these lines, all types of mutations resulted in frameshift and a premature termination of *CsWOX3* translation (Fig. 4A). Notably, on the ovary surface of *Cswox3^CRISPR^* lines, the size of fruit spines base was significantly larger compared to the 6101–4 wild-type plants, particularly in the *Cswox3^CRISPR^1* line (Fig. 4B). Subsequently, we generated *CsWOX3* overexpression lines, *CsWOX3^DOE^1* and *CsWOX3^DOE^4*, which exhibited stable GFP fluorescence and ectopically increased *CsWOX3* transcript level (Fig. 4F; Fig. S1D–E). *CsWOX3^DOE^* lines exhibited a slight decrease in the size of fruit spines base compared to wild-type plant (Fig. 4C).

Next, we employed scanning electron microscope (SEM) and quantitative statistics to assess the effects of *CsWOX3* knockout and overexpression on the average diameter of the fruit spines base (Fig. 4D–E). The results showed a significant 2-to-3-fold increase in the average diameter of fruit spines base when *CsWOX3* was knocked out, while the average diameter in the *CsWOX3^DOE^* lines decreased by 17% compared to the 3661 wild-type plants (Fig. 4G–H). These results demonstrate that *CsWOX3* plays a negative role in the morphogenesis of cucumber fruit spines. Decreased *CsWOX3* expression leads to an increase in the diameter of fruit spines base, while increased expression has the opposite effect.

### *CsWOX3* orchestrates the expression of genes involved in auxin signaling and protein ubiquitination

**Figure 5:**
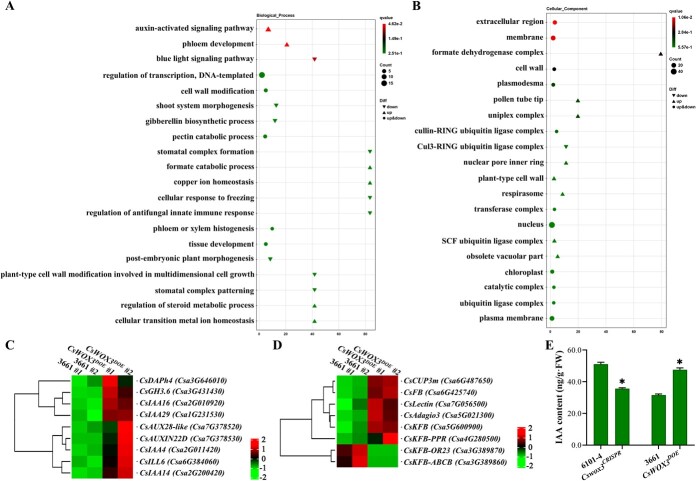
Transcriptome analysis of the *CsWOX3* overexpression lines. GO enrichment analysis of differentially expressed genes identified from transcriptome profiling of *CsWOX3* overexpression lines. GO terms are presented as biological processes (**A**) and cellular component (**B**). The *x*-axis represents GeneRatio, indicating the proportion of genes annotated in a specific item relative to all differentially expressed genes. The *y*-axis lists each GO annotation item. The point size represents the number of differentially expressed genes annotated in the pathway, and the point color represents the *q* value of the hypergeometric test. Differentially expressed genes were identified using the criteria of Fold Change ≥2 and FDR <0.01 (*N* = 3). Heatmap showing differentially expressed genes related to auxin signaling (**C**) and ubiquitin ligases (**D**). **E** Determination of endogenous IAA content within *CsWOX3* overexpression (*CsWOX3^DOE^*) and knockout (*Cswox3^CRISPR^*) lines. Significance compared to WT was determined by Student’s *t* test; ^*^P < 0.05; *N* = 3.

To further elucidate the gene regulatory networks associated with *CsWOX3* in the control of fruit spine morphogenesis in cucumber, we performed transcriptome profiling on both the 3661 line and *CsWOX3* overexpression lines. A total of 309 differentially expressed genes (DEGs) were identified, including 224 up-regulated genes and 85 down-regulated genes (Table S2, see online supplementary material). Subsequently, we performed Gene Ontology (GO) analysis on the DEGs to understand their functional classification in biological processes. We found that 245 genes were enriched. In terms of biological processes, the enrichment was mainly observed in the regulation of transcription and DNA-templated processes, auxin-activated signaling pathway, and cell wall modification (Fig. 5A). In terms of cell components, the enrichment was mainly observed in the integral component of membrane, nucleus, membrane and plasma membrane, with several ubiquitin ligases being enriched (Fig. 5B). Additionally, the enrichment was mainly observed in metal ion binding, heme binding, sequence-specific DNA binding and iron ion binding within molecular function (Fig. S2, see online supplementary material).

We conducted a heatmap analysis on the transcription levels of DEGs involved in auxin-activated signaling pathway and the enriched ubiquitin ligases (Fig. 5C–D). Our results revealed that all the auxin-related genes (*Csa1G231530*, *Csa2G010920*, *Csa2G011420*, *Csa2G200420*, *Csa3G431430*, *Csa3G646010*, *Csa6G384060*, *Csa7G378520*, and *Csa7G378530*) showed up-regulated expression (Fig. 5C). Subsequently, endogenous IAA levels were quantified within the transgenic lines of *CsWOX3* (Fig. 5E). The overexpression lines of *CsWOX3* displayed elevated auxin concentrations, while the knockout lines demonstrated reduced levels of auxin. Furthermore, among the selected genes encoding ubiquitin ligases containing F-box domains, *Csa4G280500*, *Csa5G021300*, *Csa5G600900*, *Csa6G425740*, *Csa6G487650*, and *Csa7G056500* exhibited up-regulated expression, while *Csa3G389860* and *Csa3G389870* were down-regulated (Fig. 5D). In conclusion, these findings suggest that the overexpression of *CsWOX3* leads to transcriptional changes in a set of related genes. *CsWOX3* is likely involved in the morphogenesis of fruit spines through the auxin-related pathway and ubiquitination modification pathway in cucumber.

### The conserved SPL-WOX-IAA/AUX module controls the development of cucumber fruit spines

**Figure 6:**
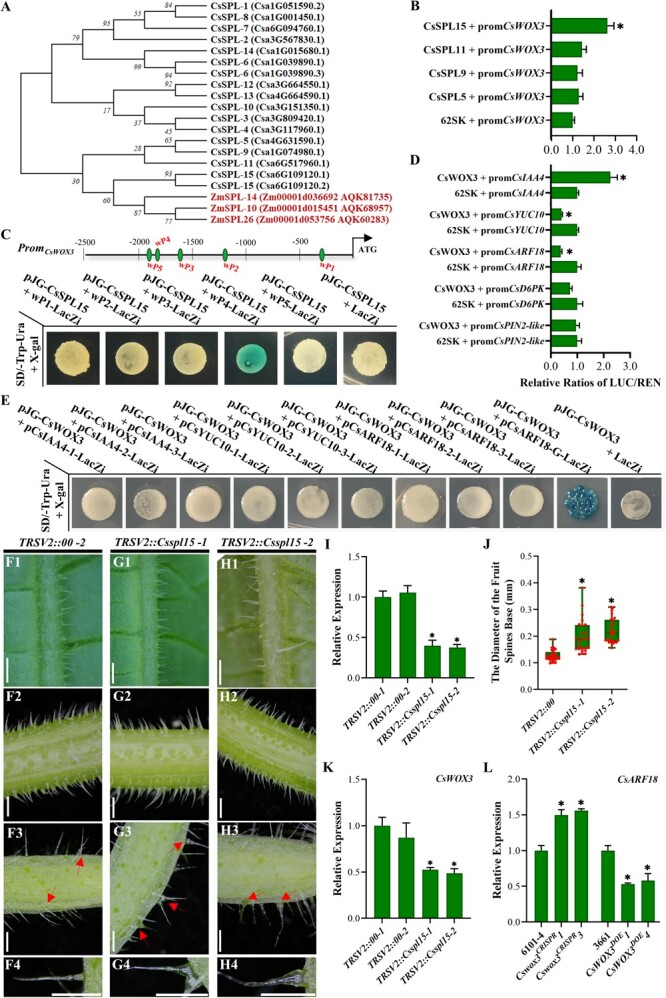
Conserved CsSPL15-CsWOX3-IAA/AUX module in the development of cucumber fruit spines. **A** Phylogenetic analysis of SPL family proteins in cucumber using maximum likelihood (ML). **B** Dual-luciferase reporter assay verification of the interaction between partial SPL proteins and CsWOX3. Error bars represent *SD* from 3 biological replicates; ^*^P < 0.05. **C** Verification of interaction between CsSPL15 and some SBP-box DNA *cis*-elements presented in the promoter of *CsWOX3* using yeast-one-hybrid LacZi reporter assay. **D** Dual-luciferase reporter assay validation of the interaction between CsWOX3 and the promoters of genes involved in auxin signaling. Error bars represent *SD* from 3 biological replicates; ^*^P < 0.05. **E** Verification of interaction between CsWOX3 and some G-box like or TAAT-box DNA *cis*-elements presented in the promoter of partial auxin-related genes using yeast-one-hybrid LacZi reporter assay. Within two independent *CsSPL15* TRSV-VIGS lines (*TRSV2::Csspl15–1* and *− 2*)**,** the phenotypic observation of trichomes attached to the surface of abaxial leaf (**F1**, **G1** and **H1**), petiole (**F2**, **G2** and **H2**) and ovary (**F3**, **G3** and **H3**), and the details of cucumber fruit spines (**F4, G4 and H4**). Bar = 1 mm. Based on 6101–4 wild-type plants, TRSV2 empty vector was used as a negative control (*TRSV2::00*), and *TRSV2::Cspds* was used as a positive control. Red arrows indicate these differences. **I** The mRNA transcriptional level of *CsSPL15* within TRSV-VIGS lines. Significance compared to *TRSV2::00* was determined by Student’s *t* test; ^*^P < 0.05; *N* = 2, *n* = 3. **J** Average diameter of cucumber fruit spines base within TRSV-VIGS lines. Significance compared to *TRSV2::00* was determined by Student’s *t* test; ^*^P < 0.05; N = 2, *n* = 25. **K** The relative transcript level of *CsWOX3* within *CsSPL15* TRSV-VIGS lines. Significance compared to *TRSV2::00* was determined by Student’s *t* test; ^*^P < 0.05; *N* = 2, *n* = 3. **L** The relative transcript level of *CsARF18* within *CsWOX3* transgenic lines. Significance compared to WT was determined by Student’s *t* test; ^*^P < 0.05; *N* = 3.

Three homologous SQUAMOSA PROMOTER BINDING PROTEIN-LIKE (SBP, known as SPL) transcription factors, ZmSPL10/14/26, were identified in maize, and mutations in these proteins led to the complete absence of trichomes [37, 38]. These proteins could bind and activate the expression of *ZmWOX3A*, the homolog of *OsWOX3B*, to orchestrate the expression of downstream auxin-related genes. This transcriptional module played a critical role in determining the fate of epidermal hair precursor cells on maize leaves. Similarly, the homolog of *OsWOX3B*, *CsWOX3*, was also involved in the development of cucumber fruit spines through auxin-related pathways. We hypothesized that the SPL-WOX-IAA/AUX transcription module may also be conserved in cucumber. To test this hypothesis, we conducted a phylogenetic analysis of all 15 cucumber SPL family proteins. The results revealed that CsSPL15 (Csa6G109120), CsSPL11 (Csa6G517960), CsSPL9 (Csa1G074980), and CsSPL5 (Csa4G631590) were closely related to ZmSPL10/14/26 and belonged to the same branch (Fig. 6A; Fig. S3A, see online supplementary material). Subsequent dual-luciferase reporter assays demonstrated that only CsSPL15 could bind and activate the expression of *promCsWOX3::LUC* (Fig. 6B). We identified five SBP-box DNA *cis*-elements (containing the conserved core sequence GTAC) that could potentially be bound by CsSPL15 within the *CsWOX3* promoter. A yeast one-hybrid LacZi assay showed that CsSPL15 indeed bound to the SBP-box (TTTGTACTT) located 1823 bp upstream of the *CsWOX3* promoter (Fig. 6C).

Furthermore, we analysed the promoter regions of various genes involved in the auxin-related pathway, including YUCCA family genes, AUX/LAX family genes, PIN family genes, ARF genes, and other related genes. We specifically focused on the genes containing more G-box like or TAAT-box DNA *cis*-elements and conducted a dual-luciferase reporter assay for verification (Fig. 6D). The results revealed that CsWOX3 could suppress the expression of *CsARF18* (Csa6G445210) and *CsYUC10* (Csa2G302220), while promoting the expression of *CsIAA4* (Csa2G011420). However, CsWOX3 had no effect on *CsPIN2-like* (Csa4G064100) and *CsD6PK* (Csa1G175730). Subsequent yeast one-hybrid LacZi assay results confirmed that CsWOX3 specifically binds to the G-box like DNA *cis*-elements located within the 1000 bp upstream region of *CsARF18*’s promoter (Fig. 6E).

To evaluate the functional conservation of this transcriptional regulatory module in cucumber, we conducted a preliminary assessment of *CsSPL15*’s biological function using the TRSV-VIGS system (Fig. 6F–I). Our findings revealed an enlargement in the diameter of fruit spines base within the two silenced lines. The diameters in all silenced lines increased more than twice compared to *TRSV2::00* lines, suggesting a negative role for *CsSPL15* (Fig. 6J). Furthermore, we observed a reduced expression of *CsWOX3* within *CsSPL15* silenced lines, providing additional evidence for the activation of *CsWOX3* transcription by CsSPL15 (Fig. 6K). Moreover, the transcriptional level of *CsARF18* was found to decrease in *CsWOX3* overexpression lines and increase in *CsWOX3* knockout lines, indicating a regulatory relationship between *CsWOX3* and *CsARF18* (Fig. 6L). Taken together, we conclude that the SPL-WOX-IAA/AUX transcription module, present in monocotyledonous plants such as maize and rice, is also conserved during the development of cucumber fruit spines.

### CsWOX3 interacts with a RING-finger E3 ubiquitin ligase CsMIEL1-like

**Figure 7:**
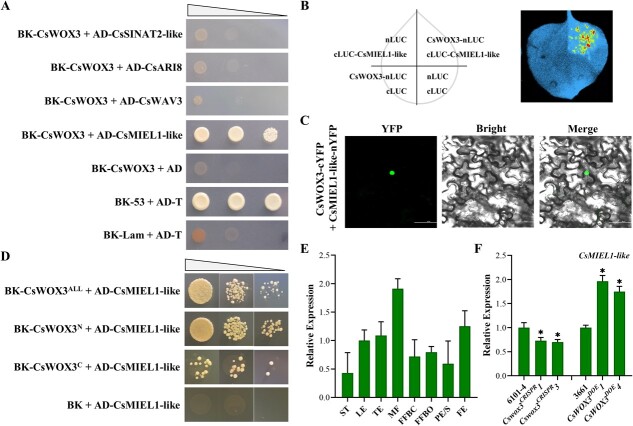
Physical interaction of CsWOX3 with the RING-finger type E3 ubiquitin ligase CsMIEL1-like. **A** Investigation of the interactions between CsWOX3 and the E3 ubiquitin ligases from the screening results using a yeast-two-hybrid assay. **B** Verifying the interaction between CsWOX3 and CsMIEL1-like through a firefly luciferase complementation imaging assay after transient *Nicotiana benthamiana* infection. **C** Bimolecular fluorescence complementation assay is used for verifying the interaction between CsWOX3 and CsMIEL1-like in *Nicotiana benthamiana* leaf cells 50 h post inoculation. Bar = 100 μm. **D** Verification of interaction between different protein domain truncations of CsWOX3 and CsMIEL1-like. **E** The relative transcript level of *CsMIEL1-like* in various cucumber tissues. FE, fruit flesh of the female floral ovary only; FFBC, female flower bud corolla; FFBO, female flower bud ovary; LE, leaf; MF, male flower; PE/S, the exocarp of female floral ovary containing fruit spines and glandular trichomes; ST, stem; TE, tendril. Error bars represent *SD* from three biological replicates. **F** The relative transcript level of *CsMIEL1-like* within transgenic lines of *CsWOX3* (*Cswox3^CRISPR^* and *CsWOX3^DOE^* lines). Significance compared to WT was determined by Student’s *t* test; ^*^P < 0.05; *N* = 3.

We previously showed that CsWOX3 functions as a transcriptional repressor and may control the cucumber fruit spine development through a ubiquitination modification pathway (Fig. 5). To comprehensively investigate the molecular mechanisms underlying *CsWOX3*’s role in negatively regulating fruit spine morphogenesis in cucumber, we performed a yeast two-hybrid library screening. Our aim was to identify potential interaction partners that collaborate with CsWOX3 in this process. Through this screening, we identified four RING-finger type E3 ubiquitin ligases and revealed that only the E3 ubiquitin ligase CsMIEL1-like (Csa7G394010) could interact with CsWOX3 (Fig. 7A; Table S3, see online supplementary material). This interaction was further confirmed through firefly luciferase complementation imaging assay and bimolecular fluorescence complementation assay (Fig. 7B–C). Additionally, we analysed the interaction between different truncated CsWOX3 protein domains and CsMIEL1-like. The results indicated that CsMIEL1-like could interact with the N-terminal HD domain of CsWOX3 (Fig. 7D). Consistent with this, five ubiquitination modification sites within the CsWOX3 HD domain were predicted using the BDM-PUB website (http://bdmpub.biocuckoo.org/prediction.php) (Fig. 1C).

CsMIEL1-like was a C_3_H_2_C_3_ type RING-finger E3 ubiquitin ligase, and primarily located in the nucleus and membrane (Fig. S3B–C). *CsMIEL1-like* exhibited high expression levels in cucumber male flowers and flesh, while displaying an opposite expression trend to *CsWOX3* within female floral organs (Fig. 7E). Notably, the transcriptional expression of *CsMIEL1-like* appears to be positively correlated with the expression of *CsWOX3* (Fig. 7F). An increase in *CsWOX3* expression led to a corresponding increase in *CsMIEL1-like* expression, while knockout of *CsWOX3* resulted in decreased expression of *CsMIEL1-like*. These findings suggested that there may be a crosstalk between *CsWOX3* and *CsMIEL1-like*. Collectively, our results suggest that CsWOX3 physically interacts with the RING-finger type E3 ubiquitin ligase CsMIEL1-like through the N-terminal HD domain.

## Discussion

Cucumber trichomes exhibit a non-branched and multicellular structure and can be found on various tissues such as leaves, flowers, and fruits [5, 17–19]. The fruit spines that adhere to the ovary’s surface can be classified into eight different structural types, and their presence and density significantly impact the visual quality of cucumber fruits [2, 5, 18–21]. In contrast, rice trichomes can be classified into three types: large hairs, micro hairs, and glandular hairs; however, these trichomes are generally considered undesirable in rice production and breeding [33, 35]. *OsWOX3B* (*NUDA-GL1*/*DEP*/*GLR1*/*LSY1*/*GLAG5*) was involved in the development of rice trichomes and interacted with the AP2/ERF protein *OsHL6* [32–36]. This interaction enhanced the binding affinities between OsHL6 and downstream auxin-related genes, thereby regulating the initiation and elongation of trichomes. Similar findings had also been confirmed in maize [37–39].

In this study, we identified a transcription factor CsWOX3 in cucumber, which was a homolog of OsWOX3B and belonged to the WUS sub-family of the WOX family (Fig. 1). CsWOX3 contained an N-terminal HD domain and a C-terminal WUS domain but lacked the typical acidic region, EAR motif, and nuclear localization sequence (NLS). Nonetheless, CsWOX3 was localized in the nucleus and exhibited strong transcriptional inhibitory activity (Fig. 2). After all, the WUS domain and the EAR motif possess transcriptional inhibition effect, while the acidic region may have a transcriptional activation effect [40–44]. Furthermore, our study confirmed that CsWOX3 binds to the G-box like DNA *cis-*element within the promoter of downstream genes, rather than the TAAT-box or TGAA-box *cis-*element bound by other WUS sub-family proteins (e.g., AtWUS) (Fig. 2B) [45–47].

Subsequently, the expression pattern of *CsWOX3* was investigated in this study (Fig. 3). It was found that *CsWOX3* was highly expressed in the younger female floral organs, particularly in the exocarp of the ovary. This expression pattern is similar to that of *OsWOX3B* in rice and *ZmWOX3A* in maize. In rice, *OsWOX3B* exhibited higher expression in young tissues, while in maize, *ZmWOX3A* was localized in the early leaf micro-trichomes and their precursor cells [32–37]. Given that WOX family proteins are well-known early regulators critical for plant growth and development, we hypothesize that *CsWOX3* may similarly control the growth and development of cucumber ovary epidermal cells [40, 48–51]. Further investigation of *CsWOX3* expression pattern using β-glucuronidase staining analysis or fused GFP protein tags is warranted in future studies.

We conducted a comprehensive investigation on the biological function of *CsWOX3*, which negatively influenced the morphogenesis of fruit spines in cucumber (Figs 3 and 4; Fig. S1, see online supplementary material). Although the size of fruit spines base in both the *Cswox3^CRISPR^* lines was significantly larger than that in the 6101–4 wild-type plants, the mutation of *CsWOX3* did not affect the fate of fruit spines in fruit cucumber genotype 6101–4 plants, which naturally aged and fell off during the later developmental stage. Interestingly, *CsWOX3* seem to affect the density of epidermal trichomes upon cucumber leaves (Fig. S1F–G, see online supplementary material). Knockout of *CsWOX3* decreases leaf trichomes density, whereas overexpression increased which density. The role of *CsWOX3* in controlling leaf trichomes mirrors that of *SlWOX3B* in tomatoes, yet this function is not applicable to cucumber fruit spines [52]. Overall, *CsWOX3* serves as a negative regulator of the development of fruit spines in cucumber. However, in *Arabidopsis thaliana*, previous studies have not reported the role of *AtWOX3* in trichomes development, unlike rice and maize. *AtWOX3*/*AtPRS* not only controlled floral organ development but also redundantly influenced leaf development with *AtWOX1* and *AtWOX5* [50, 53–55]. Although these WOX3 proteins share a homologous phylogenetic relationship, their biological functions vary among different species due to gene subfunctionalization. Moreover, WOX proteins exhibit not only redundancy and complementarity in their biological functions but also the differentiation and specific expression of their promoters. These factors contribute to a diverse range of biological functions [41, 51, 56]. Therefore, it is essential to further investigate the functions and molecular mechanisms of WOX family genes, considering the intricate nature within this gene family.

Overexpression of *CsWOX3* in cucumber resulted in transcriptional changes in a set of related genes. Gene Ontology (GO) analysis of these differentially expressed genes (DEGs) revealed enrichment of auxin-activated signaling pathway and ubiquitin ligases containing the F-box domain, suggesting that *CsWOX3* may regulate the morphogenesis of cucumber fruit spines through the auxin signaling pathway and ubiquitin-mediated proteolysis (Fig. 5; Fig. S2, see online supplementary material). Additionally, differences in endogenous IAA content were confirmed within *CsWOX3* transgenic lines, and an E3 ubiquitin ligase interacting with CsWOX3 was identified.

Plant auxin is a critical endogenous hormone involved in organ morphogenesis, including the development of plant trichomes [57, 58]. For instance, *SlARF3* was involved in the formation of epidermal cells and trichomes, while auxin transportation mediated by *GhPINs* played a vital role in the initiation and development of cotton fibers [59, 60]. In our study, we confirmed the conservation of the SPL-WOX-IAA/AUX transcription module in cucumber (Fig. 6). CsSPL15 was found to bind and activate the expression of *CsWOX3*, thereby suppressing the expression of downstream auxin-related genes such as *CsARF18*. Although we initially investigated the biological function of *CsSPL15* using TRSV-VIGS system, a more in-depth examination is warranted to elucidate the molecular mechanisms of this regulatory module, and the potential miRNA156-SPL-WOX-IAA/AUX transcription cascade should also be investigated.

As a member of the early auxin response gene family in plants, Aux/IAA proteins, a short-lived nuclear protein that can be degraded through the 26S proteasome pathway, can bind to the auxin response factor ARF proteins through the C-terminal protein domains [61]. The ARF protein family is a typical transcription factor family, with class A ARFs potentially acting as transcription activators, while class B and C ARFs are generally considered transcription inhibitors [62]. Within the canonical auxin signaling pathway, once the concentration of free auxin in plants reaches a certain threshold range, it triggers the formation of protein co-receptors involving TIR1/AFB and Aux/IAA proteins. This interaction leads to the degradation of Aux/IAA proteins via the SCF-type E3 ubiquitinprotein ligase pathway. Consequently, more ARF proteins are released, ultimately influencing the transcriptional expression of downstream genes [63, 64]. Our study reveals a novel auxin regulatory pathway mediated by *CsWOX3*. Through overexpression of *CsWOX3*, auxin synthesis is enhanced via a specific pathway, resulting in the establishment of a high-threshold auxin environment within plants. The release of ARF proteins, such as CsARF18, might be triggered, with CsWOX3 playing a key regulatory role in maintaining the optimal concentration of ARF proteins to effectively regulate plant growth and development. Additionally, CsWOX3, functioning as a regulator, is tightly controlled by the RING-finger type E3 ubiquitin ligase CsMIEL1-like. While our study provides insights into the interaction between CsWOX3 and *CsARF18*, further research is warranted to fully elucidate the intricate molecular regulatory mechanisms involving CsWOX3, CsARF18, auxin, and cucumber fruit spines.

Protein ubiquitination is a critical post-translational modification in plants, and the E3 ubiquitin ligases involved in this process can be categorized into different subfamilies, such as RING-finger ligases and cullin-RING ligases (CRLs) [65–67]. In this study, we successfully identified a RING-finger type E3 ubiquitin ligase CsMIEL1-like as a CsWOX3 interaction partner (Fig. 7). CsMIEL1-like may ubiquitinate CsWOX3 by interacting with the HD domain of CsWOX3. Interestingly, we observed that *CsMIEL1-like* exhibited a contrasting expression pattern to *CsWOX3* within the cucumber female floral organs, suggesting a crosstalk between *CsWOX3* and *CsMIEL1-like*. Based on these findings, we hypothesize that CsMIEL1-like may interact with CsWOX3 to maintain its gradient distribution, which is essential for a precise control of fruit spine morphogenesis. Further research is needed to determine if there is a negative feedback loop or dosage effect within this regulatory mechanism.

Numerous crucial genes have been identified in cucumber spine development, yet limited evidence exists linking *CsWOX3* to these genes, despite their analogous phenotypes. In our study, we have discovered a novel regulating pathway where *CsWOX3* negatively controls the morphogenesis of fruit spines in cucumber. Although we have made initial progress in understanding the potential mechanism, further extensive research is still required to gain a thorough understanding of the regulatory mechanism of *CsWOX3*. Such in-depth insights into this process could significantly advance our knowledge of cucumber trichomes development and establish a robust theoretical foundation for cucumber breeding.

## Conclusion

*CsWOX3*, a member of the WUS subclass of the WOX family, functioned as a transcriptional repressor in cucumber. *CsWOX3* exhibited a relatively high expression level in the cucumber ovary and played a negative role in the morphogenesis of fruit spines in cucumber. On one hand, CsSPL15 directly bound to and activated the expression of *CsWOX3*, thereby controlling the expression of downstream auxin-related genes. On the other, the RING-finger type E3 ubiquitin ligase CsMIEL1-like might ubiquitinate CsWOX3 through interacting with the HD domain of CsWOX3. In sum, *CsWOX3* negatively regulated the morphogenesis of cucumber fruit spines via both the auxin-related and ubiquitination pathways.

## Materials and methods

### Plant materials

The inbred cucumber lines in this experiment include North China cucumber genotype 3661 (Xintai-Mici) and fruit cucumber genotype 6101–4, which were mainly used for gene expression determination and genetic transformation. Within the fruit cucumber genotype variety 6101–4, non-glandular fruit spines (type III or IV fruit trichomes) are moderately distributed on the surface of young fruit (ovary), with smaller-sized spines base that will fall off during the later commodity period. In contrast, North China cucumber genotype variety 3661 (Xintai-Mici) has dense and large-sized spines base non-glandular fruit spines (type II fruit trichomes) that remain attached to the fruit (ovary) surface without falling off.

Cucumber seeds were soaked in 55°C warm water and germinated at 28°C for 3 days, and then the germinated seeds were cultured in 28°C/16 h light and 18°C/8 h dark environment. *Nicotiana benthamiana* was used for subcellular localization and protein expression in this experiment. The seeds were sown for about 2 weeks and then transplanted and cultured. The whole growth process was maintained under conditions of 25°C, with a photoperiod of 16 hours light and 8 hours dark. Subsequent experiments were carried out when the plants had developed 5 to 8 leaves.

### Protein sequence alignment and phylogenetic analysis

To obtain the protein sequence information of the target proteins, we accessed data from the Cucurbit Genomics database (http://cucurbitgenomics.org/organism/2) and NCBI (https://www.ncbi.nlm.nih.gov/). Subsequently, MEGA X software was used for protein phylogenetic analysis, and Jalview software was used for protein sequence alignment.

### Subcellular localization assay

*CsWOX3* or *CsMIEL1-like* CDS (without the termination codon) was cloned and recombined into pSUPER1300 plasmid between SmaI and SpeI sites, and the fusion plasmid was transformed into *Agrobacterium tumefaciens* strain GV3101. Then the *Agrobacterium* was resuspended with infection buffer (10 mM MES, 20 mM AS, 10 mM MgCl_2_, pH 5.6). The resulting solution was used to transiently infect the leaves of *Nicotiana benthamiana*. Following a 12-hour period in darkness, the plants were returned to a normal environment. After 60 hours from the injection of the *Agrobacterium* solution, GFP fluorescence was observed with a Nikon A1 confocal microscope (Japan) under 488 nm excitation light.

The subcellular localization of *CsWOX3* in cucumber protoplasts followed our previous method. After seed sterilization, the peeled cucumber seeds were germinated in 1/2 MS medium and cultivated under weak light conditions for 7 ~ 10 days. Cucumber cotyledons or young leaves were cut into filaments and then placed in an enzyme solution (cellulase R10 and macerozyme R10; Yakult Honsha, Tokyo, Japan) at room temperature. The cucumber tissue was gently shaken at room temperature for 4 to 6 hours, filtered through a 70 μm cell strainer to remove impurities, leaving only protoplasts, and then the fusion plasmid was transfected into cucumber protoplasts using the PEG4000 stress method. Finally, the protoplasts were placed under Nikon A1 confocal microscope (Japan) to observe GFP fluorescence at 20 h after transfection.

### Verification of transcriptional activation activity of CsWOX3

Various CsWOX3 domain truncations were integrated into the pGBKT7 vector between EcoRI and SalI sites. The fusion plasmids were transformed into yeast strain AH109 (or Y2H Gold) using PEG/LiAc transformation method, and its transcriptional activation activities were verified in SD/−Trp, SD/−Trp-His and SD/−Trp-His-Ade deficient medium. Subsequently, the protein truncations were fused with VP16 and verified by yeast experiments again, or the *GAL4* upstream activation sequence was serially recombined into the pGreenII-0800 vector and verified by GAL4-UAS system in *N. benthamiana* [74].

### Total RNA extraction, cDNA reverse transcription and quantitative reverse transcription PCR

In order to detect the expression of each gene in different cucumber tissues, stems, young leaves, tendrils, male flowers, female flower bud (1.0 cm) and flowering female flowers (7.0 cm) were collected. Total RNA was extracted with Huayueyang Quick RNA isolation Kit (Beijing, China), and reverse transcribed into cDNA with Tiangen FastKing RT Kit (Beijing, China). Finally, quantitative reverse transcription PCR was performed with the Cowin Biotech Ultra SYBR Mixture (Low ROX) kit (Jiangsu, China). Experiments were performed with three biological replicates and three technical replicates. The data was analysed using the 2^-ΔΔCT^ method, and Cucumber *CsTubulin* served as the internal control. The whole process was performed under RNase-free environmental conditions.

### mRNA *in situ* hybridization assay

A specific 194 bp sequence within the *CsWOX3* mRNA was identified as a hybridization probe. The high-quality hybridization probe was obtained using Roche DIG RNA Labeling kit (SP6/T7) for transcription *in vitro*. Samples were collected from the ovaries of cucumber female floral organs on the flowering day. The assay was carried out according to the steps of sample fixation, sectioning and hybridization. The whole process was performed under RNase-free environmental conditions [4, 5].

### Virus-induced gene silencing assay

Transient gene silencing was achieved using the tobacco ringspot virus-based (TRSV)-VIGS system via the *Agrobacterium*-mediated vacuum infiltration. The specific fragment of *CsWOX3* or *CsSPL15* was identified by NCBI and recombined into pTRSV2 vector (SnaBI). Cucumber line 6101–4 seeds were used in this experiment. After 2 ~ 3 days of germination under sterile conditions, the seedings were mixed with the *Agrobacterium* solution (EH105 strain), vacuumed at −900 kPa for 10 min, and co-cultured at 25°C for 3 days before planting. Finally, the seedlings were grown under the conditions of 20°C with a 16 h light/8 h dark cycle. Before phenotypic identification, qRT-PCR detection was performed on individual plants, and only plants with a silencing efficiency exceeding 60% were selected for further experiments [5, 68, 69].

### Cucumber transformation

For our gene editing experiments, we used the PKSE402-GFP vector for CRISPR/Cas9 editing. The *CsWOX3* overexpression vector, driven by a modified dual-35S promoter, was created based on the pBI121-GFP vector backbone between NheI and ApaI restriction enzymes. These recombinant plasmids were subsequently introduced into *Agrobacterium* strain EHA105. We employed cucumber lines 6101–4 and 3661 for the transformation process.

The cucumber seeds without coat were sterilized under sterile conditions and then germinated in SGM medium (MS solid medium, 2 mg/L 6-BA). After 40 hours, the excised cotyledons were infected with the *Agrobacterium* solution, resuspended in liquid IM medium (MS liquid medium, 2 mg/L 6-BA, 1 mg/L ABA, 200 μM AS, MES 1.25 mM pH 5.2), and co-cultured in solid IM medium for 3 days. The explants were then transferred into SRM medium (MS solid medium, 2 mg/L 6-BA, 1 mg/L ABA, 300 mg/L Timentin) for 30 days, and the presence of GFP fluorescent buds was detected using a LUYOR-3260 GFP fluorescent flashlight (LUYOR, USA) [70, 71].

### Yeast one-hybrid LacZi reporter system assay

According to the results of Busch *et al.* and Sloan *et al.*, three DNA *cis-*element motifs (TGAA, TAAT, and G-box like) that might be bound by CsWOX3 were verified by yeast one-hybrid LacZi reporter system. These motifs were ligated into pLacZi vectors (EcoRI and XhoI) by SolutionI ligase [46, 47].

Kong *et al.* identified the SBP-box DNA *cis-*element motifs (TNCGTACA) that could be bound by SPL family proteins. Five SBP-box *cis-*elements (wP1, −283 bp, TGTGTACTAA; wP2, −1167 bp, ACTGTACTTA; wP3, −1627 bp, TAGGTACAAG; wP4, −1832 bp, TTTGTACTTT; wP5, −1900 bp, GTAGTACAAA) containing core GTAC motif were present within the *CsWOX3* promoter. Each box motif was ligated into the pLacZi vector using EcoRI and XhoI, and the interaction between CsSPL15 and these SBP-box *cis*-elements was verified by yeast one-hybrid LacZi reporter system [37, 72, 73].

Through promoter sequence alignment analysis, it was discovered that *CsIAA4*, *CsYUC10*, and *CsARF18* contain TAAT-box DNA *cis*-elements. Additionally, *CsARF18* also contains a G-box like DNA *cis*-element. Each box motif was ligated into the pLacZi vector using EcoRI and XhoI, and the interaction between CsWOX3 and these auxin-related genes was verified by yeast one-hybrid LacZi reporter system.

### Dual-luciferase reporter assay

The CDS of *CsSPL15* or *CsWOX3* was recombined into pGreenII-62SK vectors between BamHI and HindIII as effector vectors. The full-length (2.5 kb) of *CsWOX3* promoter or the full-length (2.0 kb) of putative downstream auxin-related genes’ promoter was recombined into the pGreenII-0800 vector between BamHI and HindIII as a reporter vector. The fusion plasmid was transformed into *Agrobacterium* strain GV3101 (pSoup-P19), and *N. benthamiana* leaves were collected after infection to calculate the relative ratio of fluorescence. All the interaction between CsSPL15 and the full length of *CsWOX3* promoter, or between CsWOX3 and the full-length of putative downstream auxin-related genes’ promoter were verified by dual-luciferase reporter system.

### Yeast-two-hybrid library screening and verification of protein–protein interaction

Because CsWOX3 was a transcriptional repressor and lacked self-activation activity, the yeast mating method was used directly to screen the two-hybrid library. The fusion plasmid pGBKT7-CsWOX3 was transformed into yeast strain Y2H Gold, which would mate with the yeast library strain Y187 to form Mickey head or clover complexes. Finally, sequencing was performed on the monoclonal plaques appearing in the SD/−Trp-Leu-His-Ade medium to obtain protein sequence information. The yeast library is a cDNA library of the temporal development of cucumber cotyledon trichomes, and the library screening assay was repeated twice.

Next, the screened target protein CDS was recombined into the pGADT7 vector using EcoRI and SacI, and co-transformed with the pGBKT7-CsWOX3 fusion plasmid into the yeast strain AH109 to verify the interaction using yeast-two-hybrid assay.

### Firefly luciferase complementation imaging assay

*CsWOX3* CDS (without the termination codon) was recombined into pCAMBIA-nLUC vector between BamHI and SalI to form the CsWOX3-nLUC fusion protein. *CsMIEL1-like* CDS was recombined into pCAMBIA-cLUC vector between BamHI and SalI to generate the cLUC-CsMIEL1-like fusion protein. The VILBER Fusion FX7 multifunctional imaging system (VILBER, France) was used to measure the bioluminescence of fluorescein released after *Agrobacterium*-mediated transformation and injection into *N. benthamiana* leaves. Approximately 20 minutes before the measurement, an appropriate amount of 200 μM D-fluorescein potassium solution was injected into the *N. benthamiana* leaves and treated in dark conditions.

### Bimolecular fluorescence complementation assay

The *CsWOX3* CDS (without the termination codon) was recombined into the pSPY35S-CE vector using BamHI and SmaI to form the CsWOX3-cYFP fusion protein and the *CsMIEL1-like* CDS (without the termination codon) was recombined into the pSPY35S-NE vector between BamHI and SmaI to form the CsMIEL1-like-nYFP fusion protein. After the fusion plasmid was transformed into *Agrobacterium* strain and injected into *N. benthamiana* leaves, GFP fluorescence was observed with a Nikon A1 confocal microscope (Japan) under 488 nm excitation light.

### Transcriptome profiling and data analysis

3661 (Xintai-Mici) and *CsWOX3^DOE^* lines were used for RNA-sequencing. First, total RNA was extracted from the ovary exocarp of cucumber female flowers 2 days before flowering. In order to obtain RNA with good quality for following procedures, RNA samples were examined by NanoDrop2000 and Agient2100/LabChip GX. The qualified cDNA library was pooled and sequenced on the Illumina NovaSeq6000 sequencing platform. All the sequencing work was completed using Biomarker Technologies, and the raw data was analysed on BMKCloud (www.biocloud.net). In this project, differentially expressed genes (DEGs) were identified using the criteria of Fold Change≥2 and FDR < 0.01. Then GO and KEGG enrichment were analysed. DEGs are shown in Supplemental Table S2 (see online supplementary material).

### Determination of endogenous IAA content

We sampled the outer peel containing fruit spines of cucumber young female floral ovaries. These samples were ground into powder using liquid nitrogen, and the endogenous auxin content was determined using plate direct competition enzyme-linked immunosorbent assay (ELISA).

## Supplementary Material

Web_Material_uhae163

## Data Availability

All relevant data can be found within the manuscript and its supplemental materials.
