# Supplementary material for: The WUSCHEL-related homeobox transcription factor CsWOX3 negatively regulates fruit spine morphogenesis in cucumber (Cucumis sativus L.)
Source: Hortic Res. 2024 Jun 14;11(8):uhae163. doi: 10.1093/hr/uhae163 (PMC11298622; doi:10.1093/hr/uhae163)
Supplement: Web_Material_uhae163 [file web_material_uhae163.zip › HR-2024-280--CsWOX3--Supplemental File.docx]

**The WUSCHEL-related homeobox transcription factor CsWOX3 negatively regulates the non-glandular fruit spine morphogenesis in cucumber**

**Supplemental Information**

**Supplemental Figure S1**


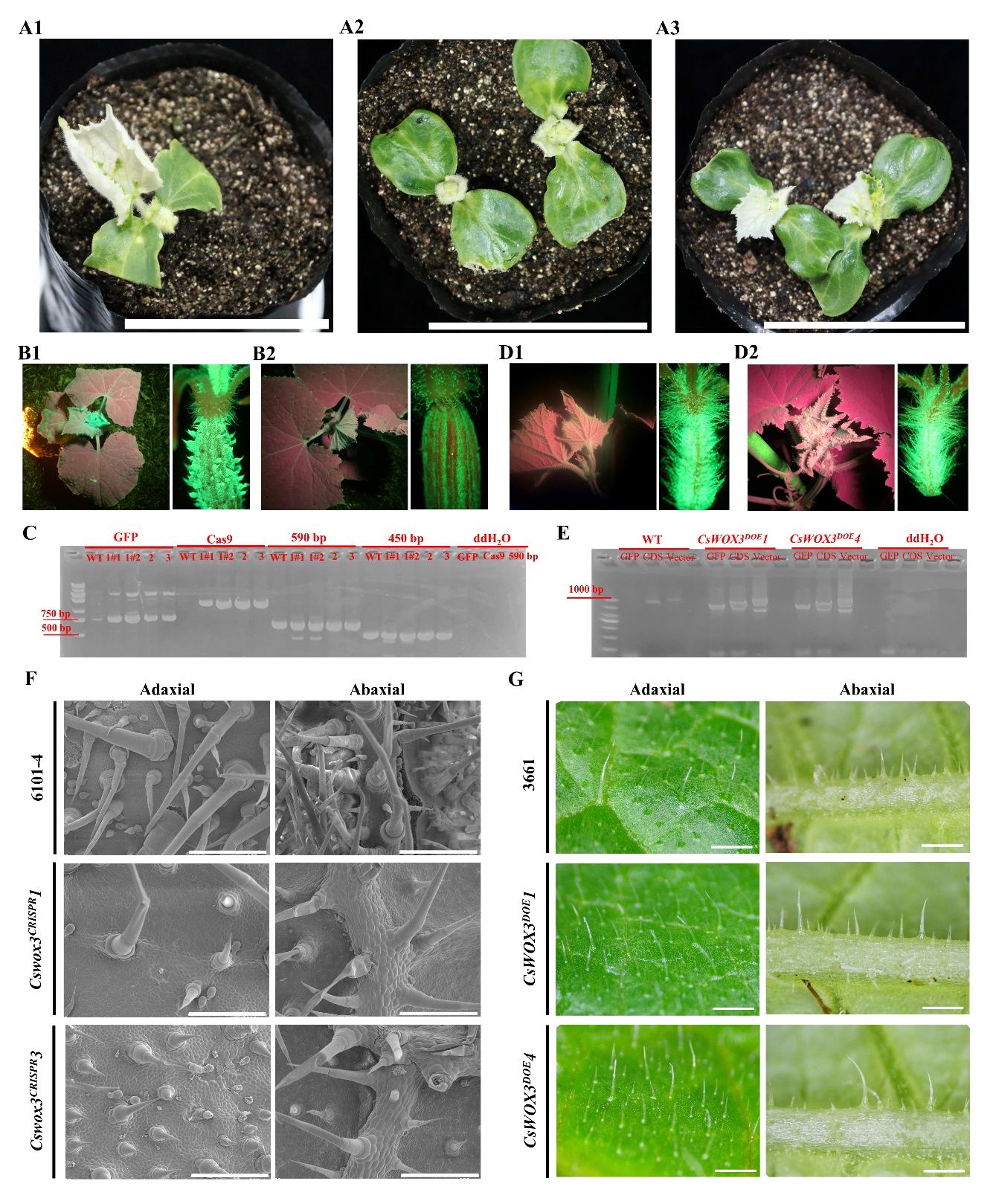


**Supplemental Fig. S1: Supplementary data for functional verification of *CsWOX3*.**

**(A)** *TRSV2::Cspds* was used as a positive control when using TRSV-VIGS transient silencing technology. Bar=5 cm.

**(B)** Stable GFP fluorescence can be observed within both T_0_ gene-edited plants: *Cswox3^CRISPR^1* (B1) and *Cswox3^CRISPR^3* line (B2). **(C)** PCR verification of *Cswox3^CRISPR^* lines. We design primers based on the sequence of vector and *CsWOX3* gene to identify each CRISPR lines. The primers bind to regions in the *GFP*, *Cas9*, and *CsWOX3*. **(D)** Stable GFP fluorescence is observed within *CsWOX3^DOE^1* (D1) and *CsWOX3^DOE^4* line (D2). **(E)** PCR verification of *CsWOX3^DOE^* lines. We designed three pairs of primers based on the vector sequence to identify the overexpression line. One pair of primers binds to the *GFP*, one pair binds to the vector skeleton sequence, and the other pair binds to the *CsWOX3* CDS regions. The phenotype of trichomes attached to the surface of adaxial and abaxial leaf within *Cswox3^CRISPR^* lines **(F**; bar=300 um) and *CsWOX3^DOE^* lines **(G**; bar=1 mm).

**Supplemental Figure S2**


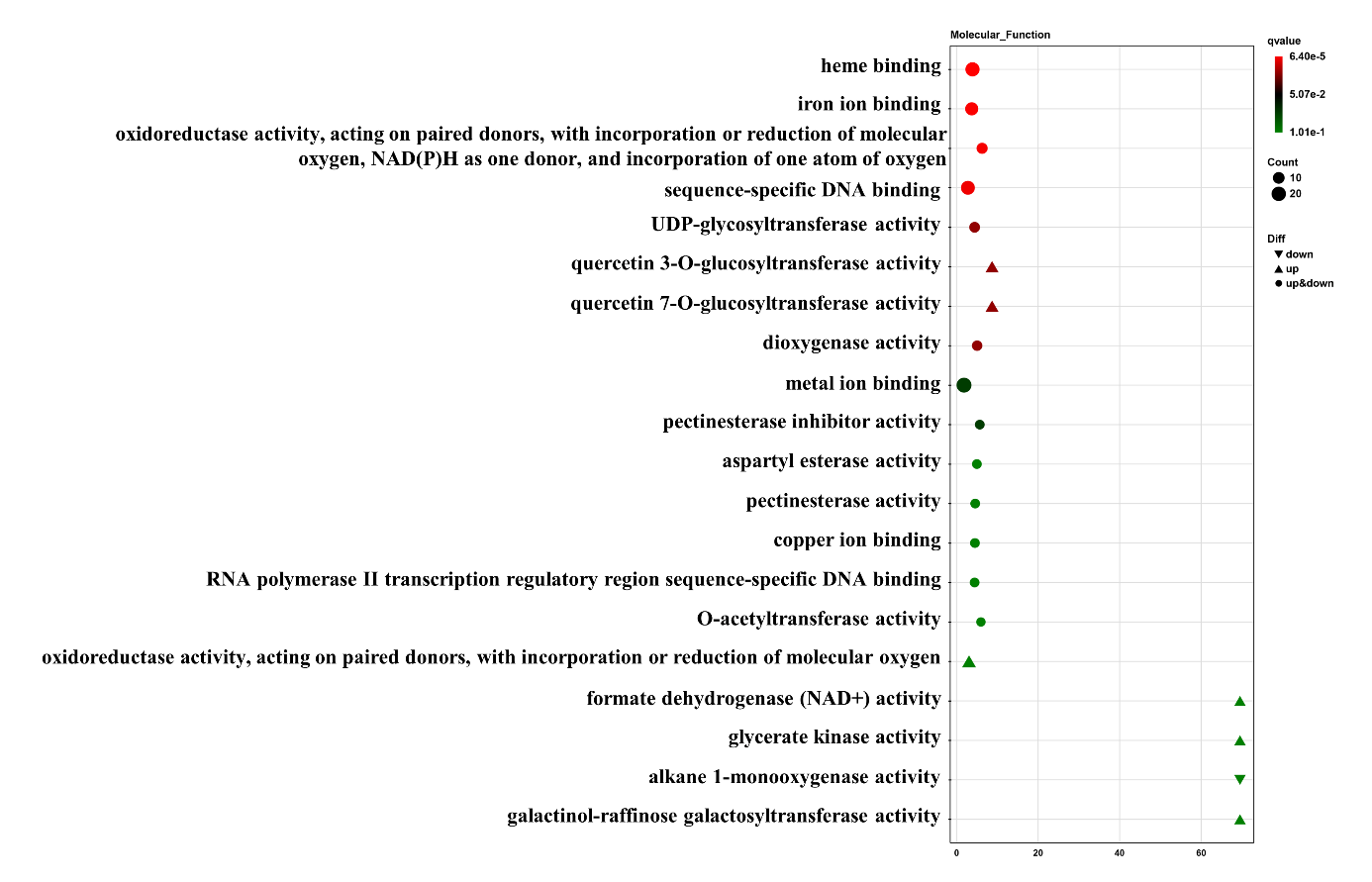


**Supplemental Fig. S2: Additional analysis of transcriptome profiling data for *CsWOX3* overexpression lines.**

GO enrichment analysis of differentially expressed genes related to molecular function in the transcriptome profiling of *CsWOX3* overexpression lines. The X-axis represents GeneRatio, indicating the proportion of genes annotated in a specific item relative to all differentially expressed genes. The Y-axis lists each GO annotation item. The point size represents the number of differentially expressed genes annotated in the pathway, and the point color represents the *q* value of the hypergeometric test. Differentially expressed genes were identified using the criteria of Fold Change≥2 and FDR<0.01 (*N*=3).

**Supplemental Figure S3**

**
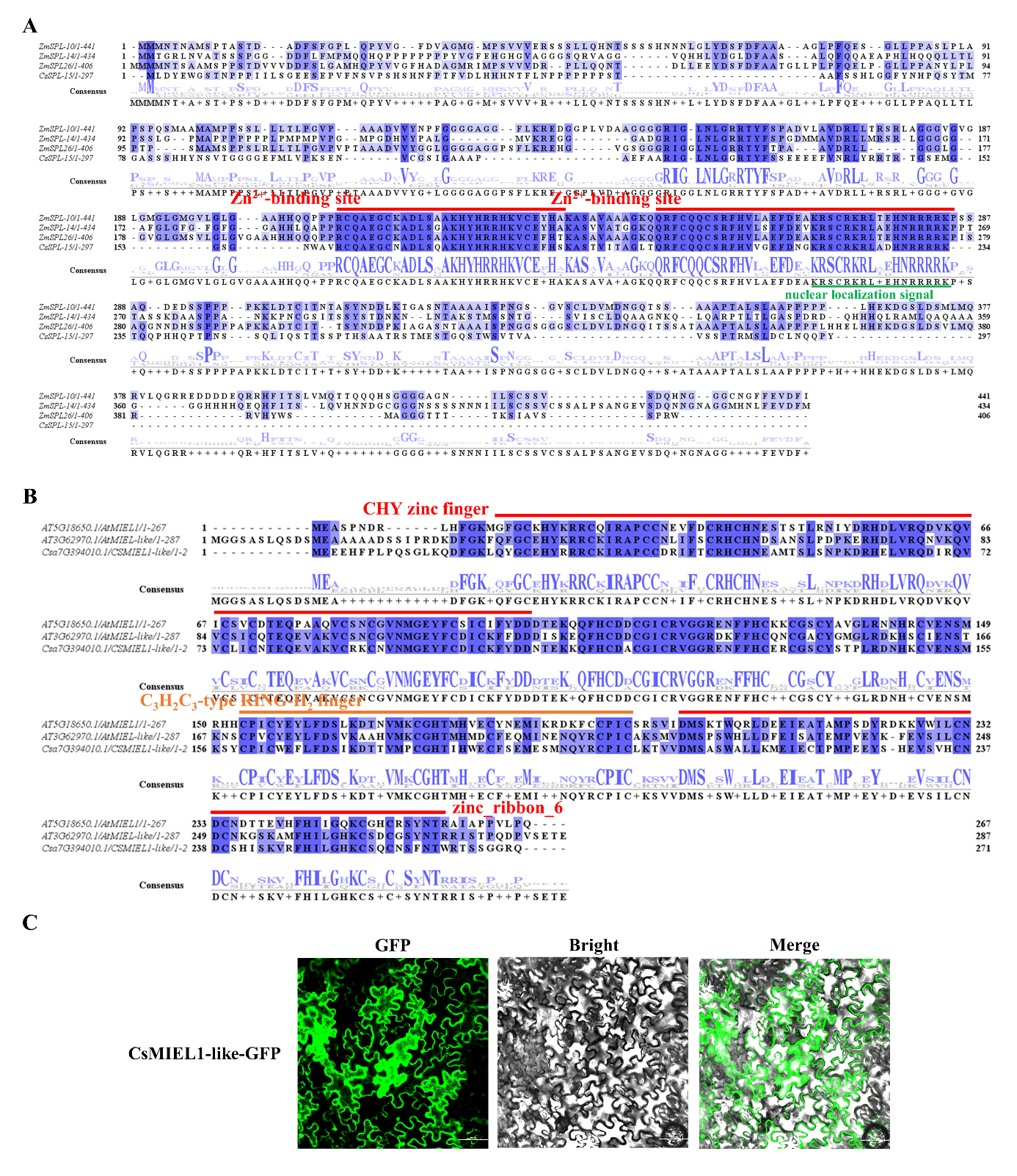
**

**Supplemental Fig. S3:** **Protein sequence alignment of CsSPL15 and CsMIEL1-like.**

**(A)** Protein domains analysis of CsSPL15. CsSPL15, as one of the SPL family proteins, conservatively contains a typical SQUAMOSA promoter binding protein domain (SPB domain; highlighted in red). **(B)** Analysis of protein sequence of CsMIEL1-like. CsMIEL1-like is a C_3_H_2_C_3_ type RING-finger E3 ubiquitin ligase that contains the CHY zinc finger, Zinc-ribbon, and C_3_H_2_C_3_ type RING-finger domains. **(C)** Subcellular localization of CsMIEL1-like in leaves cells of *Nicotiana benthamiana* 60 h after transient infection.

**Supplemental Information 1: Amino acid sequence of all WOX proteins involved in the phylogenetic analysis.**

>Csa6G505860/CsWUS

MEHPAKQQQQEQGINDQGGEGGGGSNGKGGFLCRQSSSRWTPTTDQIRILKELYYNNGVRSPSADQIQRISARLRQYGKIEGKNVFYWFQNHKARERQKKRFTSNSTSPITTSNTTTTTTDFKISSTNNNINWKSEDHSSSSHNKFPPAPSSSVMVAVGHMGNYGYGSATFENSFRECSISSGGNSSVVGYRSHNMGSWIGIDPYSSPAAAAAGGSANVFEKTKYVEESMEDHEEQEIETLPLFPIHGDRNNLGGFCSMKPEYSESYYTTTWYGRSDDNGAAGSRASLELSLNSYATISPDDGM

>Csa1G042780/CsWOX1a

MWMMGYNDAQDLNFPSDNSFNSRKLRPLIPRPLSSSSSSSHTNDFFSQFHHMATSVADQSKRELNNVLSPSSSPPVVVSSRWNPTPEQLRVLEELYRRGTRTPSADQIQHITSQLRRYGKIEGKNVFYWFQNHKARERQKRRRQMESSAITTADHSTRDLDVLERKDSGSGRTVYEEQTKNWVPSTNCSTLPEESVPIQGAAKGNVMGATDCRTENGWIQFEELHQIRNNNTNTNNFAEKDTNNIIINNNNNNINSPPSLQLLHCFPATAALRGGSQLMMNIAPITTTATVTTIMHPKCHRRSNINVISGNFDVIGVEEELEEEEEEEAECGESETLQLFPLCSDRRNGGEIVGGKNNNGCETVENSGMNSTNLTPLQFFEFLPLKN

>Csa1G025040/CsWOX1b

MWTLGYDDNGGEFCFSEATAFHGARKLRPLVPRPLLSLRANGRRPNFSSQYYHIGNVLDEERKRDQYLLNNEKSSSSSSTTSSTPIVVSSSRWNPTVEQLRILEELYRRGTRTPSADQIQHITAQLRRFGKIEGKNVFYWFQNHKARERQKRRRQTAADQTKYSEREKEEYEIEETKTKCRWPGTPTPPSTATLLSQECTETQRRRTKLVELEEEEEEETRRESVERDETWNCQLINPTRKTQHLNSVVESHNEDRGHNVVIDDINDGDKDSKAMTLQLFPLRSTKFYKYDGEREGRRDGRSRSEIMSNSDVTKSFTLSPSCSFQFFEFLPLKN

>Csa1G505930/CsWOX2

MTAEVGDGGGGVGGGGGGPPVSSRWNPTKEQISILENLYRQGVRTPSADQIQQITVRLKDYGHIEGKNVFYWFQNHKARQRQKQKQQHHHHHFNSSFPFFNHFLHFNHNNSPFPPPPPPPPPNNVVCSPYYVHQNDVGLYPQYQNNSMVIQSGGIETKRSKSERRNCIININEESTINDNNLSYQTTETLSLFPTHPTGDLQASPQSPPPPSMVPTSASDEISVDDSAHGRQPYFEFFM

>Csa6G301060/CsWOX3

MSPSSITPSRWCPTPEQVMILEEIYRNGLKTPNATQIQHITSHLSFYGKIEGKNVFYWFQNHKARDRQKLRRKLYKQLQQHHFFMKRQRFDDHQHDHHHFFQYFLPHHVPQLLPQLPSPSSLQREVGEEEAATEVEGGGGRRWMGGDEGTTVEEEDGETTCGNGTLRTLELFPVRASWVKEEEGTSGNGGWGN

>Csa2G356610/CsWOX4

MVLNMKVHQFARGFWDEPSLSLGCKRLRPLAPKLQQPTSDNTTTTTTTLTTFDLKTFIKPDCGPRKPGSSHDHSKDSSSSSSSLQTGVETHPGGTRWNPTQEQIGILEMLYSRGMRTPNAQQIEHITAQLGNYGKIEGKNVFYWFQNHKARERQKQKRNSLGLPHSPRTPPPPPIIPAFHTGEEDSPYKRKCLYWGFECLVEDNGLMCKKEEDHDRTLELFPLHPER

>Csa6G010010/CsWOX5/7

MEDGVCSRIYVKGGGGCSGESGGGGGKGSGSSKCGRWNPTAEQVKVLTELFRSGLRTPSSDQIQKISHHLSFYGKIESKNVFYWFQNHKARERQKRRKLSLPQQQHFILPPSSNLQRMFVFHLLFIPQFI

ATGGAAGATGGGGTTTGTTCTAGAATATATGTAAAAGGGGGAGGAGGATGCAGCGGAGAGAGCGGTGGTGGAGGCGGAAAAGGGAGTGGGAGTAGCAAATGTGGAAGATGGAATCCAACAGCAGAACAAGTAAAAGTACTGACAGAACTGTTCAGGTCAGGGCTTAGAACTCCGAGTAGTGATCAGATTCAGAAAATCTCTCATCATCTCAGCTTTTACGGCAAGATTGAGAGCAAGAATGTGTTTTACTGGTTTCAGAATCATAAAGCACGTGAGCGACAGAAGCGTCGCAAACTTTCTCTTCCTCAACAACAACATTTCATTCTTCCACCTTCTTCTAACCTTCAACGTATGTTTGTTTTTCACCTCCTTTTTATACCTCAATTCATTTGA

>Csa6G518270/CsWOX9

MASSNRHWPSMFKSKPCNSHHQWQHDINTTSLSSSTPTSCLRSAPYTTVGGCEERSPEPKPRWNPKPEQIRILEAIFNSGMVNPPRDEIRKIRAQLQEYGQVGDANVFYWFQNRKSRSKNKLRHIQNSKSNHNSQTTTTTSITATAATTTATCSSSSSSDKSSPKKPPIKTLTSNNSVTQNYLHQPTNEILPDPFFFPVSSTQTGSGGGSCNLSQGFCFSELCSVVQPVAEHGVGPCTSLLLSEIMSPTEALKKDLDQDKINMVKSQPQFMNFHPSNLTSSITDHTLSQSTISTPLTTLPSPTNTIPQGVGEVCGVGKSTVFINGVGFEVSSGPFNVREAFGDEAVLIHSNGQPVLTNDWGLTLHSLQHGSYYYLI

>Csa3G812740/CsWOX11

MENHGDDPNSPNQGCERAEPVRSRWTPKPEQILILESIFNSGMVNPPKDETVRIRKLLEKFGSVGDANVFYWFQNRRSRSRRRQRQLQAAATHQGSTGAIHYDCGNVSSGGYNGGSGGIMNFAGVSSSYLGGGSSSSSSSASGVGGDCSGGSGGSSGFSMSGHMGFSEVDQQMVVTSTPSFCPSETSNLEFQSGYIIIFINGVPTEVPKGAVDMKAMFGEETVLVHSSGLPVLTNEFGISLHTLQHGESYFLVSRPT

>Csa3G002330/CsWOX13a

MMEWEKPEQQNPYHHHHHNPPLCDDHLHHLTGTTAAGALYVKVMTDDQLETLRKQIAVYATICEQLVEMHKTLTAHQDLTGMRLGNMYCEPLMTSSSHKITSRQRWTPTPVQLQILERIFDQGNGTPSKQKIKEITSELGQHGQISESNVYNWFQNRRARSKRKQQSAAPAYGESEVETEVESPKDKKTKPVDFQTNQSSAPLGDDMCFQSPEMSSELHFLDPNTNKADTLFPSNGSLKTARSFSQMSFYEAGNEQLTGKIETPENYSIYQQAEGYNMTGRP

>Csa4G663700/CsWOX13b

MEWQNQQQQNQTEQLQMQVEQLSNADGVGGLYVKVMTDEQMELLRQQISVYASICEQLVEMHKAITAQQDLAGMRLGNLYCDPIMASAAGHKITARQRWTPTPVQLQILEQIFDEGNGTPSKQKIKDITLQLTQHGQISEANVYNWFQNRRARSKRKQANSLPNNADSEPETEVDSPKEKKTKPEAFQTYEHLVPKSGNMYSQRTDLSTEILSFDAQSNKGEPMFQSFGSGHTSQMTPIQNHRNNMSNDKMNVPDYSPYTPCEGYHLVE

> AT2G17950.1/AtWUS

MEPPQHQHHHHQADQESGNNNNNKSGSGGYTCRQTSTRWTPTTEQIKILKELYYNNAIRSPTADQIQKITARLRQFGKIEGKNVFYWFQNHKARERQKKRFNGTNMTTPSSSPNSVMMAANDHYHPLLHHHHGVPMQRPANSVNVKLNQDHHLYHHNKPYPSFNNGNLNHASSGTECGVVNASNGYMSSHVYGSMEQDCSMNYNNVGGGWANMDHHYSSAPYNFFDRAKPLFGLEGHQEEEECGGDAYLEHRRTLPLFPMHGEDHINGGSGAIWKYGQSEVRPCASLELRLN

>AT3G18010.1/ATWOX1

MWTMGYNEGGADSFNGGRKLRPLIPRLTSCPTAAVNTNSDHRFNMAVVTMTAEQNKRELMMLNSEPQHPPVMVSSRWNPTPDQLRVLEELYRQGTRTPSADHIQQITAQLRRYGKIEGKNVFYWFQNHKARERQKRRRQMETGHEETVLSTASLVSNHGFDKKDPPGYKVEQVKNWICSVGCDTQPEKPSRDYHLEEPANIRVEHNARCGGDERRSFLGINTTWQMMQLPPSFYSSSHHHHQRNLILNSPTVSSNMSNSNNAVSASKDTVTVSPVFLRTREATNTETCHRNGDDNKDQEQHEDCSNGELDHQEQTLELFPLRKEGFCSDGEKDKNISGIHCFYEFLPLKN

>AT5G59340.1/AtWOX2

MENEVNAGTASSSRWNPTKDQITLLENLYKEGIRTPSADQIQQITGRLRAYGHIEGKNVFYWFQNHKARQRQKQKQERMAYFNRLLHKTSRFFYPPPCSNVGCVSPYYLQQASDHHMNQHGSVYTNDLLHRNNVMIPSGGYEKRTVTQHQKQLSDIRTTAATRMPISPSSLRFDRFALRDNCYAGEDINVNSSGRKTLPLFPLQPLNASNADGMGSSSFALGSDSPVDCSSDGAGREQPFIDFFSGGSTSTRFDSNGNGL

>AT2G28610.1/AtWOX3

MSPVASTRWCPTPEQLMILEEMYRSGIRTPNAVQIQQITAHLAFYGRIEGKNVFYWFQNHKARDRQKLRKKLAKQLHQQQHQLQLQLQQIKPKPISSMISQPVNKNIIDHHNPYHHHHHNHHHNHHRPYDHMSFDCCSHPSPMCLPHQGTGVGEAPSKVMNEYYCTKSGAEEILMQKSITGPNSSYGRDWMMMMDMGPRPSYPSSSSSPISCCNMMMSSPKIPLKTLELFPISSINSKQDSTKL

> AT1G46480.1/AtWOX4

MKVHEFSNGFSSSWDQHDSTSSLSLSCKRLRPLAPKLSGSPPSPPSSSSGVTSATFDLKNFIRPDQTGPTKFEHKRDPPHQLETHPGGTRWNPTQEQIGILEMLYKGGMRTPNAQQIEHITLQLGKYGKIEGKNVFYWFQNHKARERQKQKRNNLISLSCQSSFTTTGVFNPSVTMKTRTSSSLDIMREPMVEKEELVEENEYKRTCRSWGFENLEIENRRNKNSSTMATTFNKIIDNVTLELFPLHPEGR

> AT3G11260.1/AtWOX5

MSFSVKGRSLRGNNNGGTGTKCGRWNPTVEQLKILTDLFRAGLRTPTTDQIQKISTELSFYGKIESKNVFYWFQNHKARERQKRRKISIDFDHHHHQPSTRDVFEISEEDCQEEEKVIETLQLFPVNSFEDSNSKVDKMRARGNNQYREYIRETTTTSFSPYSSCGAEMEHPPPLDLRLSFL

>AT2G01500.1/AtWOX6

MGYISNNNLINYLPLSTTQPPLLLTHCDINGNDHHQLITASSGEHDIDERKNNIPAAATLRWNPTPEQITTLEELYRSGTRTPTTEQIQQIASKLRKYGRIEGKNVFYWFQNHKARERLKRRRREGGAIIKPHKDVKDSSSGGHRVDQTKLCPSFPHTNRPQPQHELDPASYNKDNNANNEDHGTTEESDQRASEVGKYATWRNLVTWSITQQPEEINIDENVNGEEEETRDNRTLNLFPVREYQEKTGRLIEKTKACNYCYYYEFMPLKN

> AT5G05770.1/AtWOX7

MSSRGFNIKARGLCNNNNGGGGTGAKCGRWNPTVEQVKLLTDLFKAGLRTPSTDQIQKISMELSFYGKIESKNVFYWFQNHKARERQKCRKISTVKFDHRQDTDLSKPRRDNVRRHQLPAKG

>AT5G45980.1/AtWOX8

MSSSNKNWPSMFKSKPCNNNHHHQHEIDTPSYMHYSNCNLSSSFSSDRIPDPKPRWNPKPEQIRILESIFNSGTINPPREEIQRIRIRLQEYGQIGDANVFYWFQNRKSRAKHKLRVHHKSPKMSKKDKTVIPSTDADHCFGFVNQETGLYPVQNNELVVTEPAGFLFPVHNDPSAAQSAFGFGDFVVPVVTEEGMAFSTVNNGVNLETNENFDKIPAINLYGGDGNGGGNCFPPLTVPLTINQSQEKRDVGLSGGEDVGDNVYPVRMTVFINEMPIEVVSGLFNVKAAFGNDAVLINSFGQPILTDEFGVTYQPLQNGAIYYLI

> AT2G33880.1/AtWOX9

MASSNRHWPSMFKSKPHPHQWQHDINSPLLPSASHRSSPFSSGCEVERSPEPKPRWNPKPEQIRILEAIFNSGMVNPPREEIRRIRAQLQEYGQVGDANVFYWFQNRKSRSKHKLRLLHNHSKHSLPQTQPQPQPQPSASSSSSSSSSSSKSTKPRKSKNKNNTNLSLGGSQMMGMFPPEPAFLFPVSTVGGFEGITVSSQLGFLSGDMIEQQKPAPTCTGLLLSEIMNGSVSYGTHHQQHLSEKEVEEMRMKMLQQPQTQICYATTNHQIASYNNNNNNNNIMLHIPPTTSTATTITTSHSLATVPSTSDQLQVQADARIRVFINEMELEVSSGPFNVRDAFGEEVVLINSAGQPIVTDEYGVALHPLQHGASYYLI

> AT1G20710.1/AtWOX10

MEQESLNGRYGSRVMTDEQMETLRKQIAIYAVLCDQLVFLHNSLSSVPLLSSGMNPMRGEYFDPMVASSSAHGMSTRPRWTPTTTQLQILENIYKEGSGTPNPRRIKEITMELSEHGQIMEKNVYHWFQNRRARSKRKQPPTTTITSSQADDAAVTTTEERGRCGDDSGGFESYEHILFPSPDLGIEHLLNRDKFID

>AT3G03660.2/AtWOX11

MDQEQTPHSPTRHSRSPPSSASGSTSAEPVRSRWSPKPEQILILESIFHSGMVNPPKEETVRIRKMLEKFGAVGDANVFYWFQNRRSRSRRRQRQLQAAAAAADATTNTCDQTMMVSNSLPHHSGSDLGFGGCSTSSNYLFGGSSQVPSFFLGLSSSPSSCSSSSSTSSSASSSSSYGGGCDNQSNSGMENLLTMSGQMSYHEATHHHYQNHSSNVTSILCPSDQNSNFQYQQGAITVFINGVPTEVTRGGIDMKATFGEDLVLVHSSGVPLPTDEFGFLMHSLQHGEAYFLVPRQT

>AT5G17810.1/AtWOX12

MNQEGASHSPSSTSTEPVRARWSPKPEQILILESIFNSGTVNPPKDETVRIRKMLEKFGAVGDANVFYWFQNRRSRSRRRHRQLLAATTAAATSIGAEDHQHMTAMSMHQYPCSNNEIDLGFGSCSNLSANYFLNGSSSSQIPSFFLGLSSSSGGCENNNGMENLFKMYGHESDHNHQQQHHSSNAASVLNPSDQNSNSQYEQEGFMTVFINGVPMEVTKGAIDMKTMFGDDSVLLHSSGLPLPTDEFGFLMHSLQHGQTYFLVPRQT

>AT4G35550.1/AtWOX13

MMEWDNQLQPNNHHSSNLQGIDVNGGSGAGGGMYVKVMTDEQYETLRKQIAIYGTICERLVEMHKTLTAQQDLAGGRMGGLYADPMMSSLGHKMTARQRWTPTPVQLQILERIFDQGTGTPSKQKIKDITEELSQHGQIAEQNVYNWFQNRRARSKRKQHGGGSSGNNNGESEVETEVEALNEKRVVRPESLLGLPDGNSNNNGLGTTTATTTAPRPEDLCFQSPEISSDLHLLDVLSNPRDEHLVGKMGLAESYNLYDHVEDYGMSG

>AT1G20700.1/AtWOX14

MVKKKKEKEKSKEIEEMDREIQNGAYSGRVMTEEQMEILRKQIAVYAVICDQLVLLHNSLSSYHPLSSGVRPMVGGYFDPMGASSSSHRISTRHRWTPTSTQLQILESIYDEGSGTPNRRRIREIATELSEHGQITETNVYNWFQNRRARSKRKQPQTTTANGQADDVAVTTEERRSCGDSGGLESYEHILFPSPDLGIEHLLSIGKFMET

>Cucumis melo-WOX3

MSPSSITPSRWCPTPEQVMILEEIYRNGLKTPNATQIQHITSHLSFYGKIEGKNVFYWFQNHKARDRQKLRRKLYKQLQQHHFFMKRQRFDDDHDHHHFFQYFLPHHVPQLLPQLPSPSSLQRELGEEEAATEVEGGGGRRWLGGDDGTTVAEEDGETTCGNGSLRTLELFPVRGSWVKEEEGTSGNGGWGN

>Benincasa hispida-WOX3-like

MSPSGITPSRWCPTPEQVMILEEIYRNGLKTPNATQIQHITSHLSFYGKIEGKNVFYWFQNHKARDRQKLRRKLYKQLQQHHFLMKRHRFDEHTQFFHYFLPQHVPQLLPFPSPSHQKEGEEEPATEVEGGRTTWFGGDEGTTVAEEDGETTCRNGTLRTLELFPVRASWMKEEEGTSGNGGWMN

>Gossypium arboretum-WOX3

MCPAGSSRWCPTPEQVMILEEMYRSGVKTPNATQIQQITSHLSFYGKIEGKNVFYWFQNHKARERQKLRRKLTKQLQLQQQQLFHHYFDSLPSPPFQRLSYYNSPPPFPQQVGVHDAAAAAKQGMNYTWKLDVSERMDVDKSMMKIYGGDLLMMVDLSSPSLSSPCFFTTTTTGPPPLKTLELFPVTASNLKEECNKNNNG

>Oryza sativa-WOX3B

MAPAVQQQQSGGGGGSTGAAAVGSTTRWCPTPEQLMMLEEMYRGGLRTPNAAQIQQITAHLSTYGRIEGKNVFYWFQNHKARDRQKLRRRLCISHHLLSCAHYYHHHLAAAAAVVPPPQLLPPLHPSSSSSSCGGGLIDHANSLLSPTSATTPTSAAAAAAAAAYTTSYYYPFTAAAAPPPPRTSPAASPLFHYNQGGGGVVLPAAEAIGRSSSSSDYSLGKLVDNFGVALEETFPAQPQQPATTMAMTAVVDTTAVAAAAGGFCRPLKTLDLFPGGLKEEQHDVV

>Oryza sativa-WOX3

MPQTPSTRWCPTPEQLMILEEMYRSGVRTPNAAEIQQITAHLAYYGRIEGKNVFYWFQNHKARERQRLRRRLCARHQQQPSPPSSTVPPAPTAAAAGAVVQVHPAVMQLHHHHHHHHPYAAAAAAQSHHLQQQQQQQAEWPAAVDYCSTASASASATAADMAIPPCCRPLKTLELFPTKSTSGGLKEDCCSSSKSSSCSTSTN

>Oryza sativa-WOX4

MRLHHLHVAYLDHKASSSSSSPAPPSISPSSIPGSAAFPAFSFKCLRPLAPKISLPEPRKMIAPPDFVVPRARNASKLLNYTVQVPAAGTTRWNPSAEQIKVLEMLYRGGMRTPNSVQIERITEELGKYGRIEGKNVFYWFQNHKARERQKQKRAALLTLSTLDPSLLPATANETKEAPEKKEKDVEDGLASCKRRCKAWGDGAGDGDAVVATEAAGGCTDEVTLELFPLHPQGKA

> Oryza sativa-WOX7/DWT1/OsWOX9A

MASSNRHWPSMFRSKHATQPWQTQPDMAGSPPSLLSGSSAGSAGGGGYSLKSSPFSSVGEERVPDPKPRWNPRPEQIRILEAIFNSGMVNPPRDEIPRIRMQLQEYGQVGDANVFYWFQNRKSRSKNKLRSGGTGRAGLGLGGNRASAPAAAHREAVAPSFTPPPPILPAPQPVQPQQQLVSPVAAPTSSSSSSSDRSSGSSKPARATSTQAMSVTTAMDLLSPLAAACHQQMLYQGQPLESPPAPAPKVHGIVPHDEPVFLQWPQSPCLSAVDLGAAILGGQYMHLPVPAPQPPSSPGAAGMFWGLCNDVQAPNNTGHKSCAWSAGLGQHWCGSADQLGLGKSSAASIATVSRPEEAHDVDATKHGLLQYGFGITTPQVHVDVTSSAAGVLPPVPSSPSPPNAAVTVASVAATASLTDFAASAISAGAVANNQFQGLADFGLVAGACSGAGAAAAAAAPEAGSSVAAVVCVSVAGAAPPLFYPAAHFNVRHYGDEAELLRYRGGSRTEPVPVDESGVTVEPLQQGAVYIVVM

>Zea mays-WOX3B

MPQTPSTRWCPTPEQLMILEEMYRSGVRTPNAAEIQQITAHLAYYGRIEGKNVFYWFQNHKARERQRLRRRLCARHQQQYAQQQQQATAAAPASSPNSSATLLAPPAAGGSSAPCVHPAVMQLHHHHHPYATSFSMPHLGYLGQQAATVTPVLNPAAAGMVDLAGAGAGNRATGAGGAYGGGAGLYNSCSSNQLEVWDATEPMDHCNASCGAASGSSDEGGAAHLQLPACCRRPLKTLDLFPTKSTGLKDECSSSKSSSCSTSTN

>Solanum lycopersicum-Solyc06g076000.1.1

MSDSSVDMITSGGSPVGCRWNPTKEQIDLLESLYKQGIRTPSADQIQQITGRLRAFGHIEGKNVFYWFQNHKARQRQKQKQDKFAYYNRFLHRTSVFPPPNCPNGFVVVCSPYYTAQNNLGFYQQYPAVPSMIIPGPGGFKRRATCNQETLNLCEQNNSPNANKEFITQETLNLFPLHPTGVLQEKTTIADSSSSSSTCVPRDHLSSNTTSNSVEVNCFTDLGIGASDRPQPVFNFLCGN

>Solanum lycopersicum-Solyc04g078650.2.1

MYMGSSSGSLSMKVHQFTRGFLEHEAASPSLTLGCKRLRPLAPKLNTTNNDTTTTIVTPPFDLKSFIRPESSNSPPKLAYNEDKKDSSQVESHPGGTRWNPTQEQIGILEMLYRGGMRTPNAQQIEQITAQLGKYGKIEGKNVFYWFQNHKARERQKQKRNSLGLSQSPRTPSAIVTSPLSFDTRGEVVRDEDSPYKRKCRGWTFEYMEEEQQQQDEEEIINCRENGDRTLQLFPLHPEGMR

>Solanum lycopersicum-Solyc11g072770.1.1

MPRPRWSPTPQQLMILQDLYRKGLRNPTSSQVQKITTHLSLYGKIQCKNVFYWFQNHKARDRQKLRKELMMLHKIHKNTSDDDVPHQFHTTQNTNSNLEYNFPPSTFHTLYPQSPSILVHQGEGKDTSSSSTQMMNNMGNVDFPKHCVIENGMMRTNVQGWILMMTDMGPISIPSCSSNKPLETLELFPIKATGIKE

>Solanum lycopersicum-Solyc11g072790.1.1

MGRPTRWSPTPEQLMFLEEMYRKGLRNPNATQIQSITCHLSSFGKIEGKNVFYWFQNHKARDRQKLKKKLLAQMNQQQILAQYPIDAHSTTTTTTNSNNNTLFHCPTTDQYQICPLTSTTALLQEGEIKEASSQVMTYLYPMDLSKPADQNMENCMIRPYGKDWIVMMNINPNNLPYCVNRPLKTLPLFPITTTDDLKDQTTSSTSLSL

>Solanum lycopersicum-Solyc02g083950.2.1

MEHQHNIEDGGKNSNNSFLCRQSSSRWTPTSDQIRILKDLYYNNGVRSPTAEQIQRISAKLRQYGKIEGKNVFYWFQNHKARERQKKRLIAAASATDNNNISSMQMIPHLWRSPDDHHKYNTTTTNPGVQCPSPSSHGVLPVVQTGNYGYGTLAMEKSFRECSISPPGGSYHQNLTWVGVDPYNNMSTTSPATYPFLEKSNNKHYEETLDEEQEEENYQRGNSALETLSLFPMHEENIISNFCIKHHESSGGWYHSDNNNLAALELTLNSFP

>Solanum lycopersicum-Solyc02g082670.2.1

MDWEKQQQQPPVSAPQQTAEELNGTVSGGMFVKVMTDEQMEVLRKQIAVYATICEQLVDLHKSMASQHDLAGARLGNLYCDPLVTSAGHKITGRQRWTPTPMQLQILERIFEQGNGTPTKQKIKEITSELSQHGQISETNVYNWFQNRRARSKRKQQVAATNNTESEVETEVESPNEKKTKPEDLQSSHMPTSMAEDLGYENPDVSSGMHSLDPRTSKPEPMFPSDGSSKPAASYGQMSFYGMSNPRMDQLMGKMEVPGSYHPYIHADDYNMTG

>Solanum lycopersicum-Solyc06g072890.1.1

MIIPFFDVILEFYLFHSAMDDDSSSSSQSCERNETVRSRWNPKPEQIVILESIFNSGMVNPRKDETVRIRKMLEQFGAVGDANVFYWFQNRRSRSRRRQRQIQASLSAVSNSNEEQSARSSDGGAIQFQTNFVPSSVSSSSSSLGGVVGNANDGLFLFSGPMGIDQNSSITSILCSPTDNSNLNCSSSGFITVFINGVAAQVPRGPLDMKAMFGPEDLVLYHSSGVPLPVNEYGFVVQSLQHGESYFLACPCILLYTHVTLYIL

>Solanum lycopersicum-Solyc03g096300.2.1

MGTKCGRWNPTVEQVKVLTDLFRSGLRTPTTDQIQKISSQLSFYGNIESKNVFYWFQNHKARERQKRRRKVLVDETNNDEDMRIQLDNISSNKQINSNHDTDERVIETLQLFPLNSYAETEKLRLFTEEYMKENMTFSCSIGAEMDHPTLDLRLSFFS

>Solanum lycopersicum-Solyc02g077390.1.1

MASSNRHWPSMFKSKPCNSHHHQWQHDINSSIIQQRPPCNPEERSPEPKPRWNPRPEQIRILEAIFNSGMVNPPRDEIRKIRAKLQEYGQVGDANVFYWFQNRKSRSKHKQRHLQAKAQQQHHNNNNNSSHQPIITSSSSSSDKSSPNSLTFSIGTSNVMDLLNSPTSSVNQQNYNEFLSNEQPFFFTVQPPPVVPTHDHSAGFCFQDSSTFTPHSSSSGLLLNEWMGGISTQAPNNSKKDENDKINLQSQLMSYTVTSTVSPLATTTIPTISHIQGVTVDPNDAGPTRSTVFINDVAFEVGIGPFNVREVFGEDAVLIHSSGEPLITNEWGITIQPLQHGAFYYLLRTSSIASTHHI

>Solanum lycopersicum-Solyc03g118770.2.1

MWMMGYNEGGDFNMSDSCNGRKLRPLMPRVPHAPTANPTNCLRNFHGENFIALNHHQLAMSEQNKRDFNTQLVVSSRWNPTPEQLQTLEELYRRGTRTPSAEQIQHITAQLRRYGKIEGKNVFYWFQNHKARERQKRRRQLESSANGNGNGGGGDDQSQSNCNAENAERKESGANRTVFEIEQTKHWPSPTNCSTLAEKTAAKTKAGAASAAAGATAAGVAESCRVAAAERWIPFDEGEQRRSLLAERNATWQMMHLSCSPPTINNNTNCATICSNTITTATCTPIIRSCPSTPTTIDHQTKQLFKPKDHLNIFITPFRCDQKHQNIIGDEEEEGEGNGHEAQTLELFPLRSSNDNNDENNFSDKDEIGAAANLNNNFNGSHYQFFEFLPLKN

>Zea mays-GRMZM5G805026_T01

MDWGNRTKAAAAAAAPDERAGGGEGLGGYVKVMTDEQMEVLRKQISIYATICEQLVEMHRVLTEHQDTIAGLRFSNLYCDPLIIPGGHKITARQRWQPTPMQLQILESIFDQGNGTPSKQKIKEITAELSQHGQISETNVYNWFQNRRARSKRKQAAASLPNNAESEAEADEEPLADKKPKSDRPPPPPPPIQDNTKATGALSADRVSGGTRHLDTGHDQTSGVMYGCNDSGLLRSSGSSGQMSLYENFMSNPRIDRFPAKVESSRSFPHLQQHGEGFGMFG

>Zea mays-GRMZM6G260565_T01

MRLHHFHVAYLDKAAGSPPPSSSPPSISPASHSHSSSSAATIVPLALQQYCLRPLAPKISFPEARKMVVLPEFARVRNASSRLLNCTVQVPPTTTTVGGTTRWNPSPDQIRVLEMLYRG

>Zea mays GRMZM2G038252_T01

MEWVDRTKASAAAAAAAADERAGGAEGLAGYVKVMTDEQMEVLRKQISIYATICEQLVEMHRALTEHQDTIAGIRFSNLYCDPQIIPGGHKITARQRWQPTPMQLQILENIFDQGNGTPSKQRIKEITAELSHHGQISETNVYNWFQNRRARSKRKQAASLPNNAESEAEVDEESLTDKKPKSDRSLQDNKAMGAHNADRISGMHHLDTDHDQIGGMMYGCNDNGLRSSGSSGQMSFYGNIMPNPRIDHFPGKVESSRSFSHLQHGEGFDMFG

>Zea mays-GRMZM2G028622_T01

MAANAGGGGAGGGSGSGSVAAPAVCRPSGSRWTPTPEQIRMLKELYYGCGIRSPSSEQIQRITAMLRQHGKIEGKNVFYWFQNHKARERQKRRLTSLDVNVPAAGAADATTSQLGVLSLSSPPSGAAPPSPTLGFYAAGNGGGSAGLLDTSSDWGSSGAAMATETCFLQDYMGVTDTGSSSQWPCFSSSDTIMAAAAAAARVATTRAPETLPLFPTCGDDDDDDSQPPPRPRHAVPVPAGETIRGGGGSSSSYLPFWGAGAASTTAGATSSVAIQQQHQLQEQYSFYSNSTQLAGTGSQDVSASAAALELSLSSWCSPYPAAGSM

>Zea mays-GRMZM2G069274_T01

MLSRAASRIASNRRLAVRGSSGMEWVDRTKASAAAAAAAADERAGGAEGLAGYVKVMTDEQMEVLRKQISIYATICEQLVEMHRALTEHQDTIAGIRFSNLYCDPQIIPGGHKITARQRWQPTPMQLQILENIFDQGNGTPSKQRIKEITAELSHHGQISETNVYNWFQNRRARSKRKQAASLPNNAESEAEVDEESLTDKKPKSDRSLQDNKAMGAHNADRISGMHHLDTDHDQIGGMMYGCNDNGLRSSGSSGQMSFYGNIMPNPRIDHFPGKVESSRSFSHLQHGEGFDMFG

>Zea mays-GRMZM2G122537_T02

MPSQQQQQQGQQVAAGSTRWCPTPEQLMILEDMYRGGLRTPNASQIQQITAHLACYGRIEGKNVFYWFQNHKARDRQKMRRRLCMSHHLLSCAQYYAAAAHHGHAAFLAAPPPYGHQLLSPSTTSPTPAAAAAAAAAAYGYYYPATAAFAAPASRCAGNATPPSPTTQLFHYQGGGGLVPTEALGRPEYSLGKLDNFGVALDDVVVSSTSGAVVDTMGAPPVAGFEVAPPLPAAFSCRPLKTLDLFPGGLEEEQHDVA

>Zea mays-GRMZM2G140083_T01

MPSQQQQQQAAAGSTRWCPTPEQLMVLEETYRGGLRTPNASQIQQITAHLACYGRIEGKNVFYWFQNHKARDRQKLRRMLFMSQSHHLLSCAQYYAAVLAPRHGHQLLLSPSSTSPTPPAAAAAAAYGYYYSATAFAEPASSGGGLVPAEALGRPEYSSLDNFGVALDDVVVSSASAAVEMTPPGFEVVVPPPPAAAFCRPLKTLDLFPCGLKEEQHDVA

>Zea mays-GRMZM2G108933_T01

METPQQQSAAAAAAAAHGQDDGGSPPMSPASAAAAALANARWNPTKEQVAVLEGLYEHGLRTPSAEQIQQITGRLREHGAIEGKNVFYWFQNHKARQRQRQKQDSFAYFSRLLRRPPPLPVLSMPPAPPYHHARVPAPPAIPMPMAPPPPAACNDNGGARVIYRNPFYVAAPQAPPANAAYYYPQPQQQQQQQVTVMYQYPRMEVAGQDKMMTRAAAHQQQQHNGAGQQPGRAGHPSRETLQLFPLQPTFVLRHDKGRAANGSNNDSLTSTSTATATATATATASASISEDSDGLESGSSGKGVEEAPALPFYDFFGLQSSGGR

**Supplemental Information 2: The open reading frame (ORF) sequence of the positive control protein VP16.**

ATGGCCCCCCCGACCGATGTCAGCCTGGGGGACGAGCTCCACTTAGACGGCGAGGACGTGGCGATGGCGCATGCCGACGCGCTAGACGATTTCGATCTGGACATGTTGGGGGACGGGGATTCCCCGGGTCCGGGATTTACCCCCCACGACTCCGCCCCCTACGGCGCTCTGGATATGGCCGACTTCGAGTTTGAGCAGATGTTTACCGATGCCCTTGGAATTGACGAGTACGGTGGGTAG

**Supplemental Information 3: Partial nucleic acid sequence within *CsWOX3* Overexpression vector.**

*CsWOX3* overexpression vector (driven by a modified dual-35S promoter) was constructed based on the pBI121-GFP vector backbone using NheI and ApaI.

(NOS promoter) NheI + D35S promoter + CsWOX3 + ApaI + NOS terminator.

…aacactgatagtttaaactgaaggcgggaaacgacaatctgatcatgagcggagaattaagggagtcacgttatgacccccgccgatgacgcgggacaagccgttttacgtttggaactgacagaaccgcaacgttgaaggagccactcagccgcgggtttctggagtttaatgagctaagcacatacgtcagaaaccattattgcgcgttcaaaagtcgcctaaggtcactatca*GCTAGC*TGAGACTTTTCAACAAAGGGTAATATCCGGAAACCTCCTCGGATTCCATTGCCCAGCTATCTGTCACTTTATTGTGAAGATAGTGGAAAAGGAAGGTGGCTCCTACAAATGCCATCATTGCGATAAAGGAAAGGCCATCGTTGAAGATGCCTCTGCCGACAGTGGTCCCAAAGATGGACCCCCACCCACGAGGAGCATCGTGGAAAAAGAAGACGTTCCAACCACGTCTTCAAAGCAAGTGGATTGATGTGATATCTCCACTGACGTAAGGGATGACGCACAATCCCACTATCCTTCGCAAGACCCTTCCTCTATATAAGGAAGTTCATTTCATTTGGAGAGAACACGGGGGACTCTTGACCGTGGTCAATTGAGACTTTTCAACAAAGGGTAATATCCGGAAACCTCCTCGGATTCCATTGCCCAGCTATCTGTCACTTTATTGTGAAGATAGTGGAAAAGGAAGGTGGCTCCTACAAATGCCATCATTGCGATAAAGGAAAGGCCATCGTTGAAGATGCCTCTGCCGACAGTGGTCCCAAAGATGGACCCCCACCCACGAGGAGCATCGTGGAAAAAGAAGACGTTCCAACCACGTCTTCAAAGCAAGTGGATTGATGTGATATCTCCACTGACGTAAGGGATGACGCACAATCCCACTATCCTTCGCAAGACCCTTCCTCTATATAAGGAAGTTCATTTCATTTGGAGAGAACACGGGGGACGGATCCATGTCTCCATCAAGTATTACTCCTTCGAGATGGTGTCCAACACCAGAACAAGTGATGATTTTGGAAGAGATTTATAGAAATGGGCTTAAAACTCCTAACGCCACTCAAATTCAACATATTACTTCTCATCTTTCTTTCTATGGTAAAATTGAAGGCAAAAATGTTTTCTATTGGTTCCAAAATCACAAGGCTAGAGATCGCCAAAAGCTTAGGAGGAAACTCTATAAGCAGCTTCAACAACATCATTTCTTTATGAAACGACAACGTTTTGATGATCATCAACATGACCATCATCACTTCTTTCAATATTTCCTTCCTCACCATGTTCCCCAACTACTCCCACAGCTTCCTTCTCCCTCTTCTCTTCAGAGGGAGGTGGGTGAAGAGGAGGCAGCGACGGAAGTGGAGGGTGGTGGAGGGAGGAGGTGGATGGGAGGCGATGAGGGAACGACGGTGGAGGAGGAAGATGGAGAAACAACATGTGGCAATGGAACTTTAAGGACGTTGGAGTTGTTTCCGGTGAGAGCAAGTTGGGTGAAGGAGGAAGAAGGGACGAGTGGAAACGGTGGATGGGGAAATATGGATTACAAGGATGATGATGATAAGAATATGGATTACAAGGATGATGATGATAAGAATATGGATTACAAGGATGATGATGATAAGTAATGA*GGGCCC*ggcgtccacatcaacggcgtcggcggcgactgcccaggcaagaccgagatgcaccgcgatatcttgctgcgttcggatattttcgtggagttcccgccacagacccggatgatccccgatcgttcaaacatttggcaataaagtttcttaagattgaatcctgttgccggtcttgcgatgattatcatataatttctgttgaattacgttaagcatgtaataattaacatgtaatgcatgacgttatttatgagatgggtttttatgattagagtcccgcaattatacatttaatacgcgatagaaaacaaaatatagcgcgcaaactaggataaattatcgcgcgcggtgtcatctatgttactagatcgggcctcct…

**Supplemental Information 4: Amino acid sequence of all the SQUAMOSA PROMOTER BINDING PROTEIN-LIKE proteins used in this study.**

>CsSPL-1 (Csa1G051590.2)

MASSTSGSSKRARGTHNGNTQPVSCLVDGCNSDLSNCRDYHRRHKVCELHSKTPQVTIAGLKQRFCQQCSRFHSLEEFDEGKRSCRKRLDGHNRRRRKPQTDLLPRSAAGPFSYHQGPQLLPFTSSQVFPSTTVSSHGWSSGGSDNATDSGVHHNRNPQLNFVEKQQSLFVETSDQTNSINNYKASPPLGLMHGGGGSERNKMIYDRFRIGINESDCALSLLSSPQTQIQSDHQPQQQRNASISLLHPLTHQNAFDNSSDTSSSSVQISNDTHHHHHHHPHPHPHGVLNFSGIFGISSDNPGNQSPSTLPFHWE

>CsSPL-2 (Csa3G567830.1)

MDWGWGKFSGEPIFPENKQNPPSTFSDSIGSNKGSSQESSSSHGSSKRTRLLHANQNQTCLVDGCDTDLTNCKDYHRRHRVCDSHSKTPVVMVRGEEKRFCQQCSRFHSLGEFDEVKRSCRKRLDGHNRRRRKPQPESLFMSSSDFLSNCKGPIVLQFSDQQIHHVSEEIGRSLWPVRTEGEKKSSMVPSNSSAYFSYGRGDNKELPFFLHKNGKRQSKQIVSSQLSFNQQLPNVISQGSEVDNQKPVTTDTSPEDSGCALYLLSSRTVQAHSDAGLSSLVQSHLSVPVQTHETELHFSSLSDFSGSFDSKDKPVSSQLGLEMEEESDGLFQSEAPHKFPISWE

>CsSPL-3 (Csa3G809420.1)

MEMDCSSLTESGGSSNSSPPNSSAESLNGLKFGQKIYFEDVGIGELPKSGGGSFSSSAVIASTPTKKPRGGVVQAAQPPRCQVEGCRVDLTDAKAYYSRHKVCTMHSKSPKVIVAGLEQRFCQQCSRFHQLPEFDQGKRSCRRRLAGHNERRRKPPPGSLLSTRYGRFSSSIFENSNRVGSFLMDFSAYPKLAGKDVWSTGTKASERVPGNPNVSSMGKYVPHPWQSNSENNQPELLLPVSASGTSFSGPGNPSGGECINSVTDSSCALSLLSNESWGSRNRTTTIEVNALLNAEGTLVAQTTADAATNHFPTMSWGFKGNEAPTSSFRDLTSDLGLNHQVSHALTSSFSGDVQFPHLARRPYMELGHSGAFDSTQHLHWSL

>CsSPL-4 (Csa3G117960.1)

MCLNLGKRHYFEDSNAPSLILGKRAKPPFYATTVPRCQVEGCHVALVNAKDYHRRHRVCEMHSKAPKVVVLGLDQRFCQQCSRFHVISEFDDSKRSCRRRLAGHNERRRKSSHESARNCTQAENKSMTNGIAYVPSPTGRALSLLSSKNESWANSSELSQRSSAALRELIAENRAAILARQLILDRDWHPNHGGTDDFGGGGGVGFQQQGLYCEQQHSWERMNENGGSHVTLDLMQAPSSAFGLLSVREKSKEEDEECCELWNSFNDHDPHVV

>CsSPL-5 (Csa4G631590.1)

MDAKSFEGKHNFKDKTVVDDDFEYETEEEEVGGGGGGGSNSNACNALGYFDNEKKKRGAAVSGRGKSGGSVSLPSCQAHNCAADLSEAKRYHRRHKVCEFHSKAAIVMVAGIRQRFCQQCSRFHELTEFDEAKRSCRRRLAGHNERRRKSSAESQGESTSRKGSAPQAQSKESHCRQLVEDQRSRIQMAPPGSSGYKHLHIR

>CsSPL-6 (Csa1G039890.1)

MESWNYGSQGKGFLSDEMNSSTNSPLRSKYSLLGWEFKNPCSFGDNMLLTSGAQHVENQSFGELEFPQMVGKQLPDDSVSGVLNTKTDGGRNLNLVLPTSHPLHGEEESTSKLSGSIVDSNNRDSSLIDLKLGRFIDQGDAHSSKYSKRAAISSSTESSTSQKKMRSQGVNFQTAFCQVYGCNKDLSSSKDYHKRHKVCEVHSKTAKVIVNGIEQRFCQQCSRFHLLVEFDDGKRSCRKRLAGHNERRRKPQVSFHSGRAQRLLQPYNGIGDSRFQEKTLTATSFICKDILSSGLYYPEKLGENDWCKRVKVEGKSDYNSISATSLSNRHLNVKSPLLPYDFEVQIPPFQENGTSTAPTVNMLSETTCQYSHNVGGPHIDTHPLFHQTTLSSEDFGVYDAASTIQGLSGIPDSGCALSLLSSQSQSASNHPSIVHIPRAFIMSESQSNYSMSELSEKLMGVSPQASSTGITSKFASGMSEAQMGSIPTCGSSDRTVTFQIPGRVLHRSGLANPKANISYERTPTIDLLQLSSQLQRVEHQRHSMQDSA

>CsSPL-6 (Csa1G039890.3)

MESWNYGSQGKGFLSDEMNSSTNSPLRSKYSLLGWEFKNPCSFGDNMLLTSGAQHVENQSFGELEFPQMVGKQLPDDSVSGVLNTKTDGGRNLNLVLPTSHPLHGEEESTSKLSGSIVDSNNRDSSLIDLKLGRFIDQGDAHSSKYSKRAAISSSTESSTSQKKMRSQGVNFQTAFCQVYGCNKDLSSSKDYHKRHKVCEVHSKTAKVIVNGIEQRFCQQCSRFHLLVEFDDGKRSCRKRLAGHNERRRKPQVSFHSGRAQRLLQPYNGIGDSRFQEKTLTATSFICKDILSSGLYYPEKLGENDWCKRVKVEGKSDYNSISATSLSNRHLNVKSPLLPYDFEVQIPPFQENGTSTAPTVNMLSETTCQYSHNVGGPHIDTHPLFHQTTLSSEDFGVYDAASTIQGLSGIPDSGCALSLLSSQSQSASNHPSIVHIPRAFIMSESQSNYSMSELSEKLMGVSPQASSTGITSKFASGMSEAQMGSIPTCGSSDRTVTFQIPGRVLHRSGLANPKANISYERTPTIDLLQLSSQLQRVEHQRHSMQDQ

>CsSPL-7 (Csa6G094760.1)

MYWELRGIGNMDERSMDDMKGTRVALIESSSSRLMKRSRAPGSGAQVPSCMVDGCSSDLSKCRDYHRRHKVCELHSKTPKVTICGQEQRFCQQCSRFHSLVEFDDRKRSCRKRLDGHNRRRRKPQPATMTLNAGRFLYGNQGPRFLPFGNQLLTASSDVSSSWIGMIKPENNVPLCGGNSQFDFTDRRKRLLPKSSDTQPFLNVGSSNGGNVQKVLGNGSNRFFDSDCALSLLSTPVEPGEINLSSMSQSNLIPPAHFIHSDGLGLEGDPISSGLVSDGSSDANIRCYSTFQDGPDGSSVDLFRF

>CsSPL-8 (Csa1G001450.1)

MDWNLKTPSWDFTQLEQDALKNINSIGASSNFVEHIATSGDFTVDLKLGQVSNLGTKYVVNEPGVFKMAPSPSEPSKRARGSGNGAQPLSCLVDGCVSDLSNYRDYHRRHKVCELHSKTPQVTIGGLKQRFCQQCSRFHSLDEFDEGKRSCRKRLDGHNRRRRKPQSDSLSRSILSHYQGSQLLPFSSSHVYPSTLVMNHSWRDLANNTEIDARLHVQQEPTHFPDKHNIYLGSSSNDSVHKIGKQVTFMQSDNPESSVGQPLVKTIDFSENEIGRSKMLYDGFKTPGHESDCALSLLSSLQTQASGIGFSEAEQDRHLISFLQPLDVSLGHHNSLEPINSVLNGSVSNADINCSGMFHIASNDGCNEAPPPSLPFHWQ

>CsSPL-9 (Csa1G074980.1)

MATGKADGKRRLSYYEMEEEDEEDEEEDEDEEQEIDAELGFSGDFNKKKKIFTSGGSSNKKSASAFGGAPAAAPPPPCQADNCNADLSSSKRYHRRHKVCEFHAKAPVVPVTGIDQRFCQQCSRFHGLQEFDETKRSCRKRLAGHNQRRRKSSSDLHGESST

>CsSPL-10 (Csa3G151350.1)

MENPPPSPLSHPLPDVEMDIQLPMADDSSTSLWDLGDLLDFAADDQFSFSLEQDNLPSASSHYLEIQSQTPPSNSDRIRKRDPRLTCSNFLAGRVPCACPEVDAMLEAEVAAAPGKKRARTARVGAGSVRCQVPGCEVDISELKGYHRRHRVCLRCANATAVVIDDETKRYCQQCGKFHVLSDFDEGKRSCRRKLERHNNRRRRKPVDSTPSMESDRSSMQLEDVSCDGDVGKDNILFNNQTDQKEVVHLESDDGLVTSTVCSAPDQQNNVESGLTLVGTGEAQVDGGKDNSKSLASSYCDNKSTYSSMCPTGRISFKLYDWNPAEFPRRLRLQIFEWLANMPVELEGYIRPGCIILTAFVAMPKFMWIKLLEDPTTHVHNFVVARGRPLWGRGNILVYLNNMIFHAMEGESVMKIEMDMQAPKLHYVHPTCFEAGKPMEFVACGSHLRQPKFRSLVSFGGKYLDHDQSFVLPHCQKEGNATWSDHQLFKICIPHTENDIFGPAFVEVENQSGLSNFIPILIGDSETCSEMKAIQERLDMSLLVEATGSSHDTCEHSSLRQKVYSELMMDISWLLKKPSSEPMQQIMNSSQIQRFTRLLKFLICNDSTVILGRVLEHLKIVMENVESNVGVNGSNYPDLRFFEKYLDYAGDVLQLNLRKAGNSVLHLGFLKPKGGHVSQSSSENKLVSVAPGTTLKMEPRENGHFPAVAGSISTGNVETIPLINEKLSEKITIQEHSRKSCGLQFSGVLFRRQTTLFAVTFVAVCFGVCAALVHSHKVGEFAISIRRCLIDKL

>CsSPL-11 (Csa6G517960.1)

MANSEDQGWEDMIVDDDDDDNDNEEIGFVDNERRRRSGLTSARGGGGGGRSTVARCQADGCNADLTGAKPYHRRHKVCEFHSRAAVVILAGLEQRFCQQCSRFHALSEFDDTKRSCRMRLAGHNERRRKILPDFHGQSSTN

>CsSPL-12 (Csa3G664550.1)

MEAGYGGEACQLYGMGTMDLRAAVGKRNLEWDLNDWKWDGDLFIARPLNTVESGHLSRQLFPIVSGIPLTNGGSSNSSSSCSDEANMGIEKGKREVEKRRRVTVIEDENLNDEARTLSLKVGGNGSQIVERDAGSWEGTSGKKTKLAGGNSNRAVCQVEDCGADLSNAKDYHRRHKVCETHSKASNALVANVMQRFCQQCSRFHVLQEFDEGKRSCRRRLAGHNKRRRKINPDNVVNGNSPPDEQTSSYLLLTLLRILANLHSNGSNQTTDQDLLSHLIRSLACQSSEHGGKNLSGILHEPQNLLNNGALIGKSDLVSTFLSNGPQVPLRSSKQHDTPIPETPAQAIGRGGDTPAISSIKPSTSNSPPAYSEIRDSTVGQCKMMNFDLNDAYVDSDDGMEDIERQTLPVHMGTSSLECPSWVQQDSHQSSPPQTSGNSDSASAQSPSSSTGEAQSRTDRIILKLFGKAPNDFPHVLRAQVLDWLSHSPTEIESYIRPGCVVLTVYVRQTEAAWDNLCHDLSTSFNRLLDVSDDAFWKTGWVYVRVQHQIAFVYQGQVVVDTSLPLRNNNYCRITSVNPVAVSTSKKAIFSVKGINLSQPTTRLLCAIEGKYLSQEASDESTESDDNLKAQDDSQCVTFSCSIPVVYGRGFIEVEDDGFSSSSFPFIVAEEDVCSEICSLQSALELTETCSNSGETAELEGRSNAMEFIHEIGWLFHRNQLKSRLGHLDPNENLFSLPRFKWLMEFSMDHDWCAVVKKLLDILRDGTVDAGGHPSLNLALMEMGLLHRAVRKNSRSLVELLLRYPSKVKDASSSEDSASVDGETDSFLFKPNVVGPAGLTPLHIAAGKDDSEDVLDALTNDPGMVGIEAWKSARDSTGSTPEDYARLRGHYSYIRLVQRKINKRSAAGHVVLDIPSSLSDGSWNQKQNTDFTSSRFEIGRTELKPSQQHCKLCVRKPLGCGTSSSASLVYRPAMLSMVAIAAVCVCVALLFKSSPEVLYVFRPFRWELLDYGTS

>CsSPL-13 (Csa4G664590.1)

MDDPGAQVVPPIFIHQSLTSRYTDLPSIPKKRPLSYHQGQLHPHTWNPKAWDWDSSKFLTKPSNLNNTTLDDHDDTLRLNLGGRYVEDPVSKPPKKVRPGSPASVTYPMCQVDNCKEDLSNAKDYHRRHKVCELHSKSSKALVAKQMQRFCQQCSRFHPLSEFDDGKRSCRRRLAGHNWRRRKTQPEDVTSRLTRPGSRGPPSTGNLDIVSLLTVLARAQGKNEDQSVKSLLSANSDQLIQILNKINSLPLPADLAAKLPNLENFKGKAPPQSSLQHQNKLNGNPSSPSTMDLLTVLSATLAASAPDALAMLSQKSSVSSDSEKTRSSCPSGSDLQNRPLELPSVGGERSSTSYQSPMEDSDGQVQGTRVGLPLQLFGSSPEHDAPPNLTASRKYFSSDSSNPIEERSPSSSPPLLQTLFPVQSTEETTSNGKMPIRKEVNGVEVRKPPSSNIPFELFRELDGARPNSFQTIHYQAGYTSSGSDHSPSSLNSDAQDRTGRISFKLFEKDPSQFPGTLRTQIYNWLSNCPSEMESYIRPGCVVLSVYMSMSSIAWERLEENLVLHLKSLVHSEELDFWRSGRFLVYTGRQLASHKDGKIHLNKSSKAWSNPELTSVSPLAVVSGQKTSFLLRGRNLKIPGTRIHCTSMGGYISEEVMGLSSLGLSSEGIYDEIHSRSFKVGDVSPTTLGRCFIEVENGFRGNSFPVIIADATICRELRHLESDFDEFKVPDSSLESHSSVSSQPRLRDEILQFLNELGWLFQRERFSYELDNPDFLIRRFRFLLTFSAERDFCALVKTLLDILAKKCLITDGLSMKSLEMISELQLLNRSVKRRCRQMVDLLVHYHVSGVGDSEKKYLFPPNFIGPGGITPLHLAASMADAENLVDALTNDPLEIGLECWSSQLDESGRSPQAYALMRGNHNCNELVKRKLADRKNGQVSVRIGNEIEQLEVSSGERGRVKGRSCSRCAVVAARCNRRVPGSGTHRLLHRPYIHSMLAIAAVCVCVCLFLRGSPDIGLVAPFKWENLGYGTI

>CsSPL-14 (Csa1G015680.1)

MESWSYVSEGKGCMSDEMNSPTSSLARNKDSLLGWEFKNPCSFGSTMLPTSQQVDNQGFGELVFPEMIGKQLPDNSVCDILSSKVVGGRFLNPAMNSSIAFLGEDESTSKLSSSIVDSSSRDSSFIDLKLGRFADQRDAHGYKFFKGAPILSSSESSMPSKRVRASGLNSQTYFCQVYGCNKDLSSCKDYHKRHKVCEVHSKTAKVIVNGIEQRFCQQCSRFHLLAEFDDGKRSCRKRLAGHNERRRKPQVAINSGRTGRFLQSYNGSRLQGTALTATSFICQEILPNGLLHPEKYGTSDWCRTVKIEDKNEYMPLSAVHVPSGHLHSKSLFSPYDIETQVPPFHDNGPNASATNIFKENSNQYPLSVGGVNSNSRSYFHNPSLGSEYFSVYSAASTDRLSGLSDSGCALSLLSSQTQNSSTHSSGIPIGRPLVLLDGQNHYSMSQLSEKLMGVSSQVSVSGVSNKFNSSGLNTSEGCTLGPISTPETSDAVNFEISNRIFHGSNLADPGEGGPTIDLLQLSSQLQRVEHQRQAMQVKQESNAFCCLRIT

>CsSPL-15 (Csa6G109120.1)

MLDYEWGSTNPPPIILSGEESEPVFNSVPSHSHNFPTFVDLHHHNTFLNPPPPPPPSTAAFSSHLGGFYNHPQSYTMGASSSHHYNSVTGGGGEFMLVPKSENVCGSIGAAAPAEFAARIGLNLGGRTYFSSEEEEFVNRLYRRTRTGSEMGGSGNWAVRCQAEGCNADLSQAKHYHRRHKVCEFHSTASTVITAGLTQRFCQQCSRFHVVGEFDNGKRSCRKRLADHNRRRRKTQQPHHQPTPNSSQLIQSTTSSPTHSAATRSTMESTGQSTWSVTVAVSSPTRMSLDCLNQQPY

>CsSPL-15 (Csa6G109120.2)

MLDYEWGSTNPPPIILSGEESEPVFNSVPSHSHNFPTFVDLHHHNTFLNPPPPPPPSTAAFSSHLGGFYNHPQSYTMGASSSHHYNSVTGGGGEFMLVPKSENVCGSIGAAAPAEFAARIGLNLGGRTYFSSEEEEFVNRLYRRTRTGSEMGGSGNWAVRCQAEGCNADLSQAKHYHRRHKVCEFHSTASTVITAGLTQRFCQQCSRGFYSSGQVCVFVLVVGFM

>ZmSPL-10 (Zm00001d015451, AQK68957)

MMMNTNAMSPTASTDADFSFGPLQPYVGFDVAGMGMPSVVVERSSSLLQHNTSSSSHNNNLGLYDSFDFAAAAGLPFQESGLLPPASLPLAPSPQSMAAMAMPPSSLLLTLPGVPAAADVVYNPFGGGGAGGFLKREDGGPLVDAAGGGGRIGLNLGRRTYFSPADVLAVDRLLTRSRLAGGGVGVGLGMGLGMGVLGLGAAHHQQPPPRCQAEGCKADLSAAKHYHRRHKVCEYHAKASAVAAAGKQQRFCQQCSRFHVLAEFDEAKRSCRKRLTEHNRRRRKPSSAQDEDSSPPPPKKLDTCITNTASYNDDLKTGASNTAAAAISPNGSGVSCLDVMDNGQTSSAAAPTALSLAAPPPPPLHEKDGSLDSMLMQRVLQGRREDDDDEQRRHFITSLVMQTTQQQHSGGGGAGNILSCSSVSDQHNGGGCNGFFEVDFI

>ZmSPL-14 (Zm00001d036692, AQK81735)

MMTGRLNVATSSSPGGDDFLFMPMQQHQPPPPPPPPYVGFEHGHGVAGGGSQRVAGGVQHHLYDGLDFAASALQFQQAEAPHLHQQQLLTLPSSLGPMAPPPPPPPLPMPMPVPGMPGDHVYPALGMVKREGGGADGRIGLNLGRRTYFSPGDMMAVDRLLMRSRLGGGGGAFGLGFGFGFGGAHHLQAPPRCQAEGCKADLSGAKHYHRRHKVCEYHAKASVVATGGKQQRFCQQCSRFHVLSEFDEVKRSCRKRLAEHNRRRRKPPTTASSKDAASPPANKKPNCGSITSSYSTDNKNLNTAKSTMSSNTGSVISCLDQAAGNKQLQARPTLTLGASPDRDQHHHQLRAMLQAQAAAGGGHHHHQEQHFITSLQVHNNDGCGGGNSSSSNNNIILSCSSVCSSALPSANGEVSDQNNGNAGGMHNLFEVDFM

>ZmSPL26 (Zm00001d053756, AQK60283)

MMMMNTSAAMSPPSTDVVVDDFSLGAMHQPYVVGFHADAGMRIMPSVVVDRPLLQQNTDLLEEYDSFDFAATGLLPLPFQELPGLLPPANYLPLPTPSMAMSPPSLRLLTLPGVPVPTAAADVVYGGLGGGGAGGPSFLKREHGVGSGGGRIGGLNLGRRTYFTPAAVDRLLGGGLGGVGLGMSVLGLGVGAAHHHQQQPPRCQAEGCKADLSAAKHYHRRHKVCEFHTKASAVAAAGKQQRFCQQCSRFHVLAEFDEAKRSCRKRLTEHNRRRRKPISAQGNNDHSSPPPPAPKKADTCITTSYNDDPKIAGASNTAAAISPNGGSGGGSCLDVLDNGQITSSATAAAPTALSLAAPPPPLHHELHHEKDGSLDSVLMQRRVHYWSMAGGGTTTTKSIAVSSPRW
